# Biallelic variants in *RBM42* cause a multisystem disorder with neurological, facial, cardiac, and musculoskeletal involvement

**DOI:** 10.1093/procel/pwad034

**Published:** 2023-06-09

**Authors:** Yiyao Chen, Bingxin Yang, Xiaoyu Merlin Zhang, Songchang Chen, Minhui Wang, Liya Hu, Nina Pan, Shuyuan Li, Weihui Shi, Zhenhua Yang, Li Wang, Yajing Tan, Jian Wang, Yanlin Wang, Qinghe Xing, Zhonghua Ma, Jinsong Li, He-Feng Huang, Jinglan Zhang, Chenming Xu

**Affiliations:** Institutes of Biomedical Sciences, Fudan University, Shanghai 200032, China; International Peace Maternity and Child Health Hospital, School of Medicine, Shanghai Jiao Tong University, Shanghai 200030, China; Shanghai Key Laboratory of Embryo Original Diseases, Shanghai 200030, China; International Peace Maternity and Child Health Hospital, School of Medicine, Shanghai Jiao Tong University, Shanghai 200030, China; Shanghai Key Laboratory of Embryo Original Diseases, Shanghai 200030, China; State Key Laboratory of Cell Biology, Shanghai Key Laboratory of Molecular Andrology, Shanghai Institute of Biochemistry and Cell Biology, CAS Center for Excellence in Molecular Cell Science, University of Chinese Academy of Sciences, Chinese Academy of Sciences, Shanghai 200031, China; Obstetrics and Gynecology Hospital, Institute of Reproduction and Development, Fudan University, Shanghai 200011, China; International Peace Maternity and Child Health Hospital, School of Medicine, Shanghai Jiao Tong University, Shanghai 200030, China; State Key Laboratory of Rice Biology, the Key Laboratory of Molecular Biology of Crop Pathogens and Insects, Institute of Biotechnology, Zhejiang University, Hangzhou 310058, China; Verna and Marrs McLean Department of Biochemistry and Molecular Biology, Baylor College of Medicine, Houston, TX 77030, USA; International Peace Maternity and Child Health Hospital, School of Medicine, Shanghai Jiao Tong University, Shanghai 200030, China; International Peace Maternity and Child Health Hospital, School of Medicine, Shanghai Jiao Tong University, Shanghai 200030, China; Shanghai Key Laboratory of Embryo Original Diseases, Shanghai 200030, China; Obstetrics and Gynecology Hospital, Institute of Reproduction and Development, Fudan University, Shanghai 200011, China; State Key Laboratory of Cell Biology, Shanghai Key Laboratory of Molecular Andrology, Shanghai Institute of Biochemistry and Cell Biology, CAS Center for Excellence in Molecular Cell Science, University of Chinese Academy of Sciences, Chinese Academy of Sciences, Shanghai 200031, China; School of Life Science, Hangzhou Institute for Advanced Study, University of Chinese Academy of Sciences, Hangzhou 310024, Zhejiang, China; International Peace Maternity and Child Health Hospital, School of Medicine, Shanghai Jiao Tong University, Shanghai 200030, China; Shanghai Key Laboratory of Embryo Original Diseases, Shanghai 200030, China; International Peace Maternity and Child Health Hospital, School of Medicine, Shanghai Jiao Tong University, Shanghai 200030, China; Shanghai Key Laboratory of Embryo Original Diseases, Shanghai 200030, China; International Peace Maternity and Child Health Hospital, School of Medicine, Shanghai Jiao Tong University, Shanghai 200030, China; Shanghai Key Laboratory of Embryo Original Diseases, Shanghai 200030, China; International Peace Maternity and Child Health Hospital, School of Medicine, Shanghai Jiao Tong University, Shanghai 200030, China; Shanghai Key Laboratory of Embryo Original Diseases, Shanghai 200030, China; Institutes of Biomedical Sciences, Fudan University, Shanghai 200032, China; Children’s hospital of Fudan University, Shanghai 201102, China; State Key Laboratory of Rice Biology, the Key Laboratory of Molecular Biology of Crop Pathogens and Insects, Institute of Biotechnology, Zhejiang University, Hangzhou 310058, China; State Key Laboratory of Cell Biology, Shanghai Key Laboratory of Molecular Andrology, Shanghai Institute of Biochemistry and Cell Biology, CAS Center for Excellence in Molecular Cell Science, University of Chinese Academy of Sciences, Chinese Academy of Sciences, Shanghai 200031, China; School of Life Science and Technology, Shanghai Tech University, Shanghai 201210, China; School of Life Science, Hangzhou Institute for Advanced Study, University of Chinese Academy of Sciences, Hangzhou 310024, Zhejiang, China; Obstetrics and Gynecology Hospital, Institute of Reproduction and Development, Fudan University, Shanghai 200011, China; Shanghai Key Laboratory of Embryo Original Diseases, Shanghai 200030, China; Research Units of Embryo Original Diseases, Chinese Academy of Medical Sciences (No. 2019RU056), Shanghai 200011, China; Obstetrics and Gynecology Hospital, Institute of Reproduction and Development, Fudan University, Shanghai 200011, China; International Peace Maternity and Child Health Hospital, School of Medicine, Shanghai Jiao Tong University, Shanghai 200030, China; Shanghai Key Laboratory of Embryo Original Diseases, Shanghai 200030, China; Obstetrics and Gynecology Hospital, Institute of Reproduction and Development, Fudan University, Shanghai 200011, China; International Peace Maternity and Child Health Hospital, School of Medicine, Shanghai Jiao Tong University, Shanghai 200030, China

**Keywords:** *RBM42* gene, RNA-binding protein, neurodevelopmental disorder, Au-Kline syndrome, alternative splicing

## Abstract

Here, we report a previously unrecognized syndromic neurodevelopmental disorder associated with biallelic loss-of-function variants in the *RBM42* gene. The patient is a 2-year-old female with severe central nervous system (CNS) abnormalities, hypotonia, hearing loss, congenital heart defects, and dysmorphic facial features. Familial whole-exome sequencing (WES) reveals that the patient has two compound heterozygous variants, c.304C>T (p.R102*) and c.1312G>A (p.A438T), in the *RBM42* gene which encodes an integral component of splicing complex in the RNA-binding motif protein family. The p.A438T variant is in the RRM domain which impairs *RBM42* protein stability *in vivo*. Additionally, p.A438T disrupts the interaction of *RBM42* with hnRNP K, which is the causative gene for Au-Kline syndrome with overlapping disease characteristics seen in the index patient. The human R102* or A438T mutant protein failed to fully rescue the growth defects of *RBM42* ortholog knockout ΔFgRbp1 in *Fusarium* while it was rescued by the wild-type (WT) human *RBM42*. A mouse model carrying *Rbm42* compound heterozygous variants, c.280C>T (p.Q94*) and c.1306_1308delinsACA (p.A436T), demonstrated gross fetal developmental defects and most of the double mutant animals died by E13.5. RNA-seq data confirmed that *Rbm42* was involved in neurological and myocardial functions with an essential role in alternative splicing (AS). Overall, we present clinical, genetic, and functional data to demonstrate that defects in *RBM42* constitute the underlying etiology of a new neurodevelopmental disease which links the dysregulation of global AS to abnormal embryonic development.

## Introduction

Neurodevelopmental disorders (NDDs) are a group of disorders which impair normal brain development or function characterized by the loss of cognitive, emotional, and motor developmental milestones (Askeland et al., 2022). Common NDDs include intellectual disability, attention deficit/hyperactivity disorder, autism spectrum disorder, and schizophrenia with a prevalence of ~3% worldwide (Parenti et al., 2020). Many NDDs are syndromic disorders which have multisystem involvement in addition to the neurological symptoms (Shankar et al., 2022). NDDs are genetically heterogeneous with more than 1,000 underlying loci identified (Soden et al,. 2014; Niemi et al., 2018; Tărlungeanu and Novarino, 2018). The development of next-generation sequencing technology enabled an effective genomic approach for the molecular diagnosis of NDDs and the search for new disease-causing genes (Fernandez-Marmiesse et al., 2018). However, the genetic basis remains unknown in at least half of individuals affected by NDDs (Wright et al., 2018).

In this work, we identified biallelic loss-of-function variants in the *RBM42* gene associated with a previously unrecognized NDD syndrome presenting neurological, facial, cardiac, and musculoskeletal abnormalities. The *RBM42* gene is located on human chromosome 19q13.12, which contains 10 exons encoding RNA-binding motif protein 42, or *RBM42*. This protein belongs to the RNA-binding motif (RBM) protein family with an RNA recognition motif (RRM) located on its N-terminus. RRM is evolutionarily conserved from bacteria to vertebrate which is also the most common RNA-binding domain found in 1% of all human proteins (Ciuzan et al., 2015; Corley et al., 2020). RBM protein family is a subgroup of RNA-binding proteins (RBPs) which play an important role in post-transcriptional gene regulation including mRNA production, turnover, localization, translation, and splicing (Sutherland et al., 2005; Gebauer et al., 2021). Currently, over 50 human RBM proteins have been identified in which a few are known to be associated with a Mendelian disease (Li et al., 2021). Pathogenic variants in *RBM10* cause X-linked recessive TARP syndrome characterized by talipes equinovarus, atrial septal defect, Robin sequence, and persistent left superior vena cava (Johnston et al., 2010). Cerebellar hypoplasia, cerebellar vermis hypoplasia, abnormal corpus callosum, developmental delay, and hypotonia are evident in nearly all reported TARP cases (Kaeppler et al., 2018). Thrombocytopenia-absent radius syndrome is caused by biallelic pathogenic variants in the *RBM8A* gene, which is characterized by reduction in the number of platelets and absence of the radius (Albers et al., 2012). Neurological defects include severe mental retardation, agenesis of corpus callosum, and hypoplasia of cerebellar vermis (Skórka et al., 2005).

Human *RBM42* is ubiquitously expressed in different organs while it is enriched in brain, heart, lung, liver, and peripheral blood mononuclear cells (Fishilevich et al., 2016; Consortium, 2020). *RBM42* is an integral component of the B complex, U4/U6.U5 tri-snRNP, and pre-B complex involving gene splicing (Schmidt et al., 2014; Agafonov et al., 2016; Boesler et al., 2016). It binds to tri-snRNP and stabilizes the quasi-pseudoknot via the RRM domain which primes pre-B complex for receiving the 5ʹ splice site (Charenton et al., 2019). Two *RBM42* orthologs, *Fusarium graminearum* FgRbp1 and *Toxoplasma gondii* TgRRM1, are reported to function as vital splicing factors (Suvorova et al., 2013; Wang et al., 2021). FgRbp1 is a pre-mRNA splicing regulator which binds to the motif CAAGR on its target mRNAs and interacts with the 3ʹ splice site recognition protein FgU2AF23, leading to enhanced recruitment of essential splicing factor FgU2AF23 to the target mRNAs (Wang et al., 2021). Human *RBM42* can fully rescue the growth defect caused by deletion mutant FgRbp1 (Wang et al., 2021). TgRRM1 associates with the U4/U6.U5 tri-SNP complex which is required for the assembly of spliceosome (Suvorova et al., 2013). The TgRRM1 missense mutant (c.505A>T, p.Y169N) causes G_1_ cell cycle arrest while human *RBM42* can rescue its growth and splicing defects (Suvorova et al., 2013).

It has been reported that *RBM42* interacts with hnRNP K (Fukuda et al., 2009), which is the causative gene for Au-Kline syndrome (AKS) (Au et al., 2018). The hnRNP K protein binds RNA and belongs to the heterogeneous nuclear ribonucleoprotein (hnRNP) family, implicated in chromatin remodeling, transcription, splicing, and translation (Bomsztyk et al., 2004). AKS Patients usually present with severe global development delay, intellectual disability, congenital heart disease, and typical facial abnormalities including metopic ridging, broad nasal bridge, ptosis, and downturned mouth (Au et al., 2018; Choufani et al., 2022). Importantly, the disease characteristics in AKS are highly reminiscent of those seen in the index patient with *RBM42* variants. We herein identified the genetic defect in a patient affected with a syndromic neurological disorder. Results from clinical evaluation, genetic analysis, biochemical and cellular assays, and model system studies support that *RBM42* is essential for cellular growth and normal development in human.

## Results

### Clinical characterization

**Figure 1.**
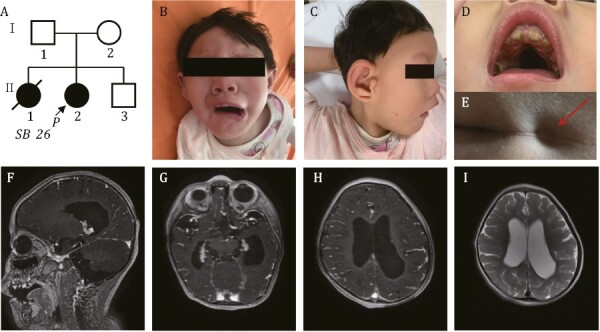
**Pedigree of the family in this study.** (A) Arrow indicates the proband. (B–D) Distinctive facial features presented by the proband. (B) Downturned “beaked” mouth of the proband (symmetric face at rest and asymmetric face while crying and laughing). (C) Metopic ridging and auricular deformity. (D) Oligodontia and narrow high arched palate were seen. (E) Red arrow indicates the sacral dimple of proband. (F–I) Brain MRI shows cerebral dysplasia, including agenesis of the corpus callosum (F), lateral ventriculomegaly (G), pachygyria (H) and decreased white matter of the lateral ventricle posterior horn (I).

The proband’s (II-2, Fig. 1A) prenatal history was notable for congenital heart disease, including ventricular septal defect, right atrial enlargement, and suspected aortic riding. At 24 weeks of gestation, her biparietal diameter was three weeks behind. The fetus also showed a mild dilatation of the posterior horn of the left lateral ventricle. Parental and fetal karyotype and chromosomal microarray results were normal. Prenatal toxic and radioactive exposure were denied. The proband was born at 41^+6^ weeks of gestation by oxytocin stimulation with a normal birth weight and length. Facial dysmorphism and multiple congenital anomalies were noted during the newborn period. Blood test showed slightly reduced platelet count 80 × 10^9^/L (normal range 100–400 × 10^9^/L). Biochemical tests were essentially normal with only slightly reduced plasma ornithine and glycine not suggestive of a metabolic disease. Mitochondrial DNA sequencing result was normal.

The proband came for examination and genetic workup when she was 2 years old. Physical examination was notable for microcephaly (<3rd percentile), low weight (<3rd percentile), and low height (<25th percentile). She was non-ambulatory, non-verbal, and dysmorphic with metopic ridging, broad nasal bridge, nystagmus, long palpebral fissures, ptosis, auricular deformity, oligodontia, malocclusion, narrow high arched palate, downturned mouth, and a sacral dimple (Fig. 1B–E). The patient had severe global developmental delay, intellectual disability, hypotonia, hearing loss, and feeding difficulties. Brain magnetic resonance imaging (MRI) demonstrated agenesis of the corpus callosum, lateral ventriculomegaly, pachygyria and decreased white matter of lateral ventricle posterior horn (Fig. 1F–I). Echocardiography confirmed ostium secundum atrial septal defects, patent ductus arteriosus, and mild tricuspid regurgitation. The differences seen in prenatal and postnatal cardiac evaluation may be resulted from the limited resolution of fetal ultrasound scan and the interpretation of transient cardiac phenotypes before birth.

The proband is the 2nd child to non-consanguineous Chinese parents. The mother had a previous elective abortion (II-1, Fig. 1A) at 26 weeks of gestation due to fetal heart defects and intrauterine growth restriction, but prenatal or postnatal diagnosis was not performed. The proband has another healthy younger brother (II-3, Fig. 1A).

### Whole-exome sequencing analyses and Sanger sequencing

**Figure 2.**
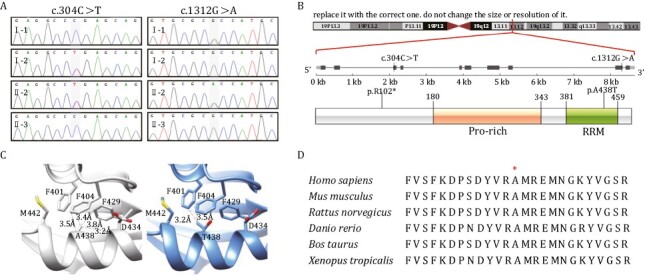
**Whole-exome sequencing identified two compound heterozygous variants in the *RBM42* gene.** (A) Sanger sequencing confirmed that the proband (II-2) inherited the *RBM42*: c.304C>T variant from her mother (I-2), and the c.1312G>A variant from the father (I-1). Her younger brother (II-3) is not affected. (B) The genomic location and the amino acid changes of the two *RBM42* variants. The 438 residue resides in the RRM domain. (C) 3D structural model of wild-type (WT) and mutated *RBM42* proteins were constructed by AlphaFold (Jumper et al., 2021). The WT hydrophobic Alanine sits in a hydrophobic pocket which consists of F401, F404, and F429, meanwhile the mutated hydrophilic Threonine may destabilize the protein by a bulkier side chain. (D) Multi-species alignment for *RBM42* proteins. The A438 amino acid is conserved across species from xenopus to human.

A clinical trio-WES test was performed on the index family to search for the underlying pathogenic variants of the proband. No pathogenic variants in known disease genes were identified which could explain the patient’s multisystem symptoms. However, two compound heterozygous variants in the *RBM42* gene (NM_024321.5), c.304C>T (p.R102*) and 1312G>A (p.A438T) were found in the proband while the mother and father were heterozygous for these two variants respectively. The whole-exome sequencing (WES) results were confirmed by targeted Sanger sequencing. The proband’s healthy younger brother does not carry any of these two familial *RBM42* variants (Fig. 2A). The c.304C>T and c.1312G>A variants have not been reported in the gnomAD database (as March 11th, 2023) (Karczewski et al., 2020). The c.304C>T change is in the third of 10 exon in the *RBM42* gene which results in a stop-gain variant leading to possible non-sense mediated decay. The c.1312G>A variant causes a missense change from alanine to threonine at the amino acid residue 438 which is localized in the conserved RRM domain of the *RBM42* protein (Fig. 2B and 2D). The hydrophobic alanine sits in a hydrophobic pocket which includes three phenylalanine residues, F401, F404, and F429 (Fig. 2C) (Jumper et al., 2021). The missense change to a hydrophilic threonine is predicted to disrupt the hydrophobic pocket by a bulkier side chain resulted in a destabilized protein (Fig. 2C) (Jumper et al., 2021). The p.A438T variant is predicted to be damaging by *in silico* analyses (VarCards) including SIFT, PROVEAN, and Mutation Taster (Kumar et al., 2009; Schwarz et al., 2010; Choi and Chan, 2015; Li et al., 2018). Phylogenetic analysis shows that *RBM42* orthologs are evolutionally conserved in multicellular organisms (Fig. S2). The RRM domain shares a high level of similarity across species and the alanine at the amino acid residue 438 is conserved from fungi to human (Fig. S3). Although *RBM42* is not known to be associated with a human disease, hnRNP K is an interacting protein of *RBM42* and linked to AKS (Au et al., 2018). Importantly, the overall disease characteristics seen in our patient are highly concordant with AKS including severe global development delay, central nervous system (CNS) defects, congenital heart disease, and typical facial features such as metopic ridging, broad nasal bridge, ptosis, abnormal helix, and downturned mouth (Table 1) (Choufani et al., 2022).

**Table 1.** Comparison of manifestations between the patient with ***RBM42*** variants and Au-Kline syndrome.

Features	Present case	Summary of 32 AKS patients
General information		
Sex	Female	15 female, 17 male
Locus	A truncating and missense variant in the *RBM42* gene	11 truncating, 14 missense, 3 splice, and 4 indel variants in the *HNRNPK* gene
Central nervous system		
Global developmental delay/intellectual disability	+	32/32
Hypotonia	+	32/32
Hypoplasia corpus callosum	+	6/24
Heterotopia	−	5/24
Spinal syrinx	−	5/21
Hyporeflexia	+	12/23
High pain tolerance	+	12/22
Congenital heart disease	+	19/32
Facial features		
Craniosynostosis	+	6/31
Metopic ridging	+	16/31
Long palpebral fissures	+	24/31
Ptosis	+	19/31
Shallow orbits/prominent eyes	+	17/32
Broad nasal bridge with hypoplastic alae nasi	+	24/32
Downturned “beaked” mouth	+	23/32
Cleft palate/high arch/broad uvula	+	8/30
Malocclusion/Open bite	+	11/25
Deep midline groove to tongue	+	16/30
Abnormal helix	+	13/32
Preauricular pits	−	5/30
Branchial defect	−	1/24
Hearing loss	+	5/30
Musculoskeletal		
Muscle weakness	+	14/27
Sacral dimple	+	14/25
Hip dysplasia	+	10/31
Joint hypermobility	−	19/30
Scoliosis	−	13/31
Pes planus	−	11/25
Inverted nipples	−	11/25
Polydactyly	−	5/31
Talipes	−	6/28
Vertebral segmentation abnormalities	−	3/29
Gastrointestinal difficulty	+	22/31
Genitourinary (GU) system		
Hydronephrosis	−	17/29
Cryptorchidism	NA	13/16

NA, not applicable.

Besides the above *RBM42* variants, a *de novo* heterozygote variant, c.274-1G>A in the *WAS* (NM_000377.2) gene, was found in the proband which may explain the patient’s mild thrombocytopenia (Fig. S1). Pathogenic variants in the *WAS* gene cause Wiskott-Aldrich syndrome, an X-linked recessive immunodeficiency disease characterized by thrombocytopenia, eczema, and recurrent infections. Female heterozygote carriers of the *WAS* pathogenic variants may have mild symptoms due to skewed X chromosome inactivation (Lutskiy et al., 2002), but it cannot explain the patient’s global development defects involving multiple systems.

### *RBM42* mRNA and protein reduction associated with the c.304C>T (p.R102*) and 1312G>A (p.A438T) variants

**Figure 3.**
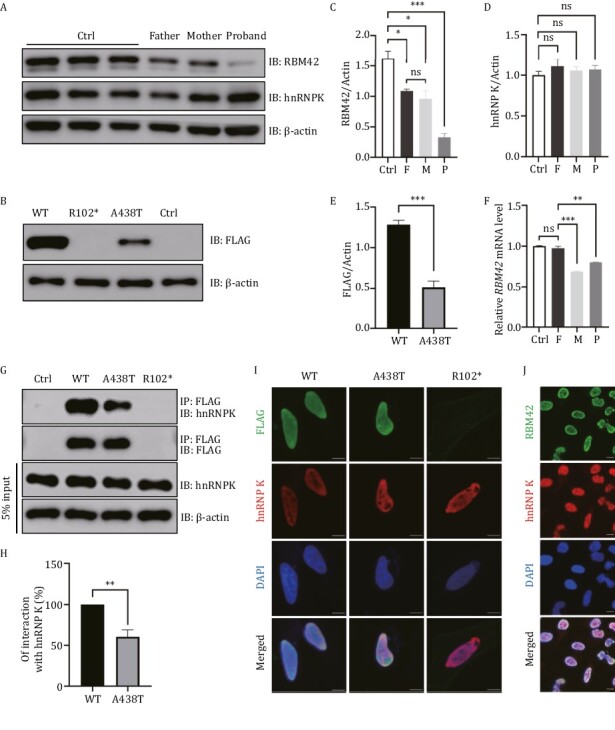
**Expression of WT and variant *RBM42* in different cell lines.** (A) The expression of *RBM42* and hnRNP K were measured in the lymphoblastoid cell lines (LCLs) of the index family and three healthy controls (Ctrl). Father: A438T/WT, Mother: R102*/WT, Proband: R102*/A438T. IB: Immunoblot. (B) 293T cells were transfected with 1 μg DNA of FLAG-tagged *RBM42*-WT, R102*, A438T or empty control (Ctrl). Expression of the transfected plasmids were detected at 48 h post-transfection by Western blot. (C) The densitometry value of endogenous *RBM42* in LCLs were normalized to the amount of β-actin. Results are the mean ± SEM of three independent experiments. (D) The densitometry value of endogenous hnRNP K in LCLs was normalized to the amount of β-actin. Results are the mean ± SEM of three independent experiments. (E) The densitometry value of overexpression FLAG in 293T cells was normalized to the amount of β-actin. Results are the mean ± SEM of six independent experiments. (F) The mRNA level of *RBM42* in peripheral blood leukocytes was measured by qRT-PCR and normalized to the expression of β-actin. Results are the mean ± SEM of three independent experiments. Using a healthy male as control (Ctrl). (G) 293T cells were transfected with 2.5 μg DNA of FLAG-tagged *RBM42*-WT, R102*, A438T or empty control (Ctrl). Immunoprecipitations were performed 48 h post-transfection and followed by Western blot analysis. (H) The densitometry values of co-immunoprecipitated endogenous hnRNP K were normalized to the amount of immunoprecipitated FLAG-*RBM42*-WT, or A438T. Results are the mean ± SEM of three independent experiments. (I) HeLacells were transfected with 1 μg DNA of FLAG-tagged *RBM42*-WT, A438T or R102*. Immunofluorescence was performed 48 h post-transfection. (J) Immunofluorescence results of endogenous *RBM42* in untransfected HeLa cells. Scale bars: 10 μm. The statistical significance was determined using an unpaired Student’s *t* test. Data are considered significant when *P* values are < 0.05 (*), < 0.01(**), or < 0.001 (***).

To explore the functional impacts of the *RBM42* variants identified above in the proband, we first set out to measure the RNA and protein levels in the index family members. The proband (0.801 ± 0.003, relative abundance mean ± standard error of the mean) and the mother (0.688 ± 0.015) both showed reduced *RBM42* mRNA compared to the father (0.975 ± 0.041) and controls (1.000 ± 0.012, Fig. 3F). The *RBM42* protein levels in the proband and parents were all reduced with the proband (0.327 ± 0.067) having the lowest level (control: 1.629 ± 0.117, father: 1.085 ± 0.034, mother: 0.959 ± 0.135, Fig. 3A and 3C). These results suggest that the c.304C>T (p.R102*) variant causes non-sense mediated decay while the 1312G>A (p.A438T) variant impairs the RMB42 protein stability. Plasmids containing mutant *RBM42* with either R102* or A438T variant were transiently expressed in 293T cells. The R102* variant had no detectable protein expression while the A438T variant protein (0.513 ± 0.078) was expressed at a remarkably low level compared to the wild-type (WT) protein (1.284 ± 0.051) suggesting that the A438T variant indeed compromised the protein stability (Fig. 3B and 3E).

It has been reported that *RBM42* interacts with hnRNP K through its C-terminus where the A438T variant is located (Fukuda et al., 2009). Co-immunoprecipitations showed that the A438T variant reduced the interaction between *RBM42* and hnRNP K while the hnRNP K protein level was not affected (Fig. 3G and 3H). Additionally, immunofluorescence showed that both endogenous and transiently expressed *RBM42* were predominantly present in the nucleus with hnRNP K (Fig. 3I and 3J). Despite its reduced affinity with hnRNP K, the A438T variant had no effects on hnRNP K subcellular localization (Fig. 3I).

### *RBM42* orthologs are functional conserved in fungi and human

**Figure 4.**
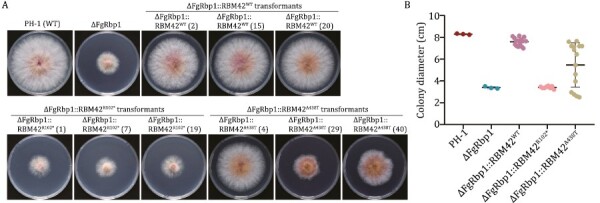
The R102* and A438T mutant human ortholog *RBM42* cannot rescue the growth defects of ΔFgRbp1 in *Fusarium graminearum* to the WT level. (A) Colony morphology of the WT strain PH-1, the FgRbp1-deletion mutant (ΔFgRbp1), and the heterologous complemented strain (ΔFgRbp1::*RBM42*^WT^, ΔFgRbp1::*RBM42*^R102*^ and ΔFgRbp1::*RBM42*^A438T^) on potato dextrose agar (PDA) at 25°C for 3 days. For each type of genetic complementation, three representative transformants are presented in the figure, distinguished by numbers in parentheses. (B) The colony diameter of the complemented strains (ΔFgRbp1::*RBM42*^WT^, ΔFgRbp1::*RBM42*^R102*^, ΔFgRbp1::*RBM42*^A438T^) and the control strains (ΔFgRbp1 and PH-1) were measured after incubating at 25°C for 3 days on PDA.

Phylogenetic analysis demonstrates that *RBM42* orthologs are evolutionally conserved in fungi (Fig. S2). The RRM domain of FgRbp1 in *F*. *graminearum*, the causal agent of *Fusarium* head blight of wheat, and *RBM42* share 51% amino acid sequence identity (Fig. S3). A previous study had showed that human *RBM42* could fully restore the growth defects in ΔFgRbp1 to the WT level, indicating FgRbp1 orthologs were functional conserved across eukaryotic kingdoms (Wang et al., 2021). To determine the functional impacts caused by the human R102* and A438T variants, the constructs expressing *RBM42*-R102* and *RBM42*-A438T variants driven by the fungal constitutive promoter RP27 were independently transformed into ΔFgRbp1 with the WT *RBM42* used as a positive control. From two independent transformation experiments, we obtained a total of 15 transformants for *RBM42*-WT complementation, eight transformants for *RBM42*-R102* complementation, and 15 transformants for *RBM42*-A438T complementation. All the resulting complemented transformants ΔFgRbp1::*RBM42*^WT^ showed similar growth level as *F*. *graminearum* WT strain PH-1, while ΔFgRbp1::*RBM42*^R102*^ had similar growth defects as ΔFgRbp1 (Fig. 4A and 4B). In the ΔFgRbp1::*RBM42*^A438T^ transformants, only part (40%) of transformants had similar growth rate as WT, while the growth of 60% transformants had not been restored to the WT level (Fig. 4A and 4B). The A438T variant exhibited a wide spread of growth curve, indicating that it was a hypomorphic variant retaining partial function of the protein as the phenotype of hypomorphic variants tends to be more variable in the presence of genetic and non-genetic modifiers (Cehajic-Kapetanovic et al., 2020; Aksentijevich and Schnappauf, 2021; Benkirane et al., 2021; Wongkittichote et al., 2023) Overall, the results in the fugal complementation assays suggest that R102* is a null allele and the A438T variant is a hypomorph allele, which both impair the mycelial growth function of *RBM42in vivo*.

### *Rbm42* pathogenic variants cause severe intrauterine developmental defects and embryonic lethality in mice

**Figure 5.**
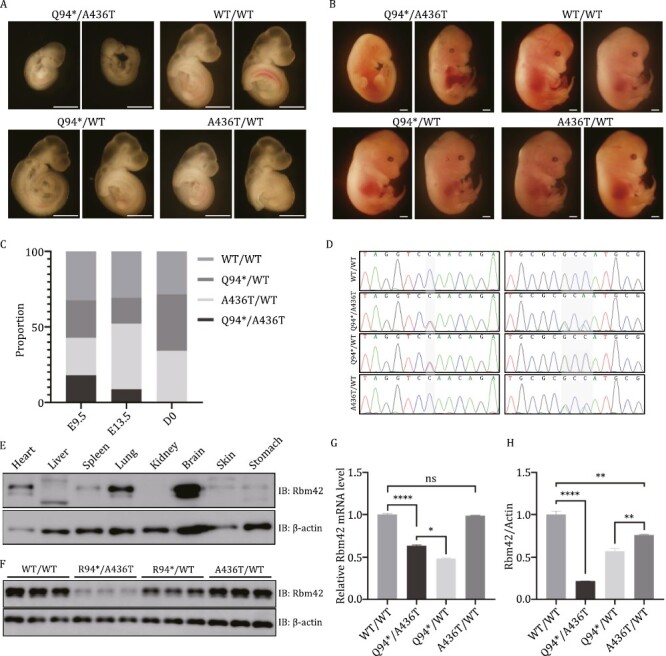
**Mouse model harboring *Rbm42* variants.** (A) Embryo morphology at E9.5. (B) Embryo morphology at E13.5. (C) The ratio of animals with different genotypes during development. (D) Genotyping of different mice by Sanger sequencing. (E) The expression of *Rbm42* protein in different organs of newborn WT/WT mice. (F) At E9.5, the expression of *Rbm42* protein was measured by Western blot in different genotype mice. (G) At E9.5, the mRNA expression of *Rbm42* was measured by qRT-PCR. Results are the mean ± SEM of three independent experiments. (H) At E9.5, the expression of *Rbm42* protein was measured by Western blot. Results are the mean ± SEM of three independent experiments. Scale bars: 1 mm. The statistical significance was determined by an unpaired Student’s *t* test. Data are considered significant when *P* values are < 0.05 (*), < 0.01(**), < 0.001 (***), or < 0.0001 (****).

To further understand the physiological impacts of the *RBM42* variants to mammalian development, mouse models harboring the c.280C>T (p.Q94*) and c.1306_1308delinsACA (p.A436T) *Rbm42* variants were established by CRISPR/Cas9. No Q94*/Q94* live-born mice were achieved and only two A436T/A436T of 25 pups were identified after the cross of A436T/WT heterozygous animals. No gross development defects in growth, motor ability, and brain structure were seen in the A436T/A436T mice. The Q94*/WT and A436T/WT strains were crossed to obtain compound heterozygous mutant mice (Fig. 5D). After genotyping 35 pups from seven litters, only one compound heterozygous mutant pup was identified, indicating severe embryonic lethality in animals with the biallelic loss-of-function variants. Next, the mutant animals were collected in early embryonic development. At E9.5, the Q94*/A436T mutants accounted for 17.9% (5/28) of the total, which dropped to 8.7% (2/23) at E13.5 and further to 0% (0/32) after birth (Fig. 5C). Notably, the Q94*/A436T embryos had abnormal morphology and development behind other litters during the same stage. No significant difference was found among embryos of Q94*/WT, A436T/WT and WT/WT (Fig. 5A and 5B). We found *Rbm42* protein in brain, heart, and lung was enriched compared to other organs of newborn WT mice (Fig. 5E). Consistent with the result in human cells, the mRNA and the protein level of *Rbm42* in the Q94*/A436T (0.635 ± 0.012, 0.215 ± 0.004) and Q94*/WT (0.484 ± 0.008, 0.569 ± 0.035) mutant mice were remarkably lower than the A436T/WT (0.986 ± 0.006, 0.760 ± 0.008) and WT/WT (1.000 ± 0.012, 1.000 ± 0.043) at E9.5 (Fig. 5F–H). These results suggest that *Rbm42* is essential for normal mouse embryonic development.

### Abnormal mRNA transcript levels caused by the *Rbm42* variants

**Figure 6.**
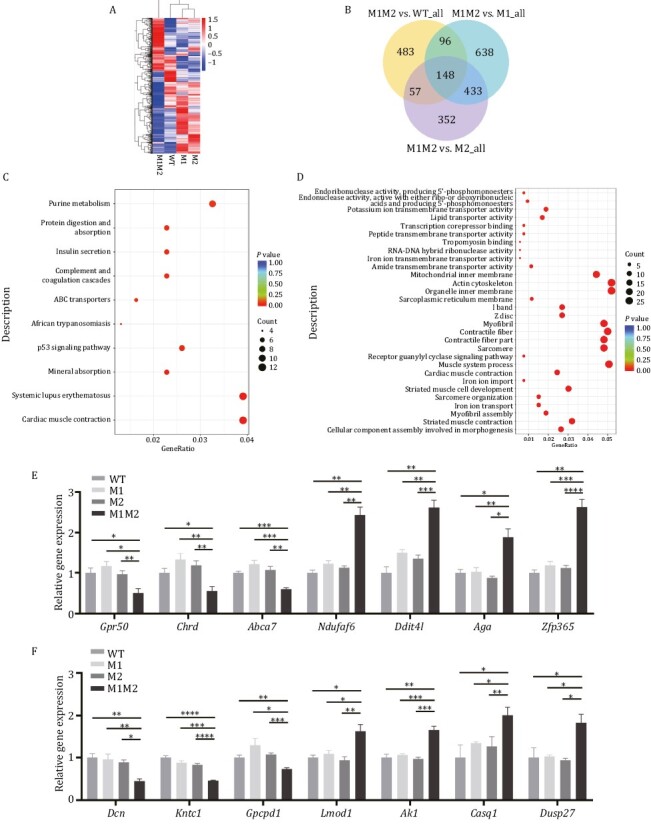
**Gene expression analysis by RNA-seq performed on E9.5 for *Rbm42* mutant mice.** (A) Heat map depicting the expression pattern of differentially expressed genes. (B) Venn diagram showing the number of genes that are shared among three pairwise (M1M2 mice to the M1, M2, and WT animals) comparisons. (C) The dotplot for Kyoto encyclopedia of genes and genomes analysis showing the top 10 terms for DEGs, compared between M1M2 and WT. (D) The dotplot for Gene Ontology term enrichment analysis including top 10 molecular function, 10 cellular component and 10 biological process, compared between M1M2 and WT. (E and F) qPCR validation for genes involved in neurological (E) or myocardiac (F) function terms among M1M2, M1, M2 and WT E9.5 mouse embryos. The data represent mean ± SEM (*n* = 3 for WT, *n* = 4 for M1, *n* = 5 for M2, *n* = 4 for M1M2). Data are considered significant when *P* values are < 0.05 (*), < 0.01(**), < 0.001 (***), or < 0.0001 (****).

To investigate whether global gene expression was perturbed by the *Rbm42* variants, RNA-seq was performed on Q94*/A436T (M1M2), Q94*/WT (M1), A436T/WT (M2), and WT/WT (WT) E9.5 mouse embryos. Heat map comparison with hierarchical clustering of the differentially expressed genes (DEGs) in M1, M2, and WT showed similar gene expression patterns (Fig. 6A). Next, we compared the M1M2 mice to the M1, M2, and WT animals as controls separately. DEGs were identified using a negative binomial distribution model with the *P*-value ≤ 0.05, and fold change ≥ 1.5. There were 148 overlapping DEGs among three pairwise (M1M2 mice to the M1, M2, and WT animals) comparisons consisting of 63 upregulated and 85 downregulated genes (Fig. 6B). Kyoto encyclopedia of genes and genomes (KEGG) analysis of RNA-seq showed that “neuroactive ligand receptor interaction” was enriched in M1M2 (Figs. S5A and S6A). Additionally, Gene Ontology (GO) showed that upregulated “dopaminergic neuron differentiation” and downregulated “negative regulation of glial cell proliferation” were also enriched in M1M2 (Fig. S4A and S4B). On the other hand, “cardiac muscle contraction” was mostly enriched in KEGG categories of M1M2 (Figs. 6C, S5A and S6A). In addition, “muscle process”, “muscle development”, and “myofibril assembly” were all highly enriched in biological process and “sarcomere”, “contractile fiber”, “myofibril”, “I band”, and “actin cytoskeleton” were differentially enriched in cellular component of M1M2 (Figs. 6D, S5B and S6B). Among the intersected DEGs, we validated three downregulated genes (*Gpr50*, *Chrd*, *Abca7*) and four upregulated genes (*Ndufaf6*, *Ddit4l*, *Aga*, *Zfp365*) involved in neurological function (Fig. 6E; Table S2). Seven deregulated genes in myocardial pathways were also validated (Fig. 6F; Table S3). Overall, the transcriptomic analyses suggest that *Rbm42* pathogenic variants may disrupt normal neurological and myocardial developments. These results are consistent with the patient’s clinical manifestations of intellectual disability, brain structure abnormality, and congenital heart defects.

### Alternative splicing analysis in E9.5 mouse embryos

**Figure 7.**
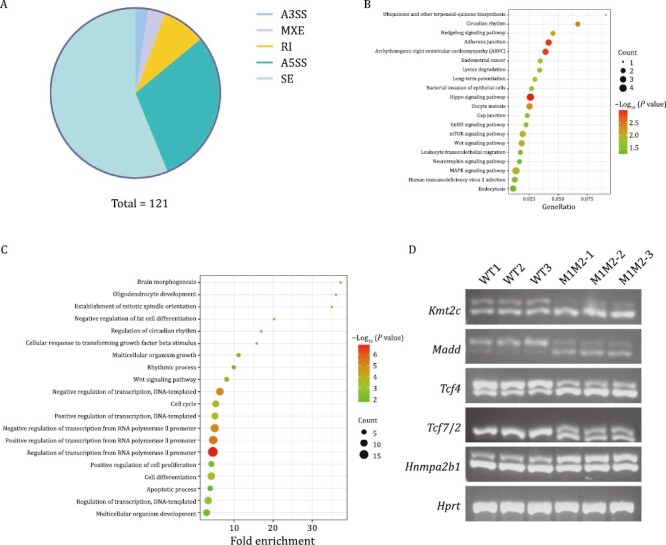
**Alternative splicing (AS) analysis by RNA-seq performed on E9.5 for *Rbm42* mutant mice.** (A) A chart summary of AS events for each type. (B) Kyoto encyclopedia of genes and genomes pathway analysis of all AS genes. (C) Gene Ontology analysis revealed enrichment of biological process involving genes that participate in AS. (D) RT-PCR validation of AS events in five genes. *Hprt* as a loading control. A3SS: alternative 3ʹ splicing site, MXE: mutually exclusive exons, RI: retained intron, A5SS: alternative 5ʹ splice site, SE: skipped exon.

As *RBM42* is a known splicing regulator (Charenton et al., 2019), the RNA-seq data was examined to investigate whether gene splicing was altered by the *Rbm42* variants. In the E9.5 Q94*/A436T embryos, a total of 121 alternative splicing (AS) events were identified involving 111 genes [False discovery rate (FDR) < 0.05, Table S4]. Skipped exon, alternative 5ʹ splice site, retained intron, mutually exclusive exons, and alternative 3ʹ splicing site accounted for 56%, 30%, 8%, 3%, and 3% of these AS events respectively (Fig. 7A). Except *Aen* and *Gorab*, the other DEGs did not show alternatively spliced species. KEGG analysis identified significant enrichment of Hippo signaling pathway in both skipped exon genes (Fig. S7A) and all AS genes (Fig. 7B), which is essential for embryonic development (Ma et al., 2019; Zheng and Pan, 2019). GO analysis showed that the biological process was enriched in the regulation of transcription RNA polymerase II promoter (Fig. 7C). In addition, the enriched cellular components were mainly localized in the nucleus (Fig. S7B), and the molecular functions were mainly associated with protein binding (Fig. S7C). Different AS of five genes in M1M2 were confirmed (Fig. 7D), including two (*Tcf7l2I* and *Hnrnpa2b1*) involving in transcription (Iwanaga et al., 2005; Moran-Jones et al., 2005; Yi et al., 2005) and three (*Kmt2c*, *Madd*, and *Tcf4*) involving in neurological functions (Zweier et al., 2007; Kleefstra et al., 2012; Schneeberger et al., 2020). Human *KMT2C*, *MADD* and *TCF4* are causative genes for three neurodevelopmental disorders, Kleefstra syndrome 2, DEEAH syndrome and Pitt-Hopkins syndrome, respectively (Zweier et al., 2007; Kleefstra et al., 2012; Schneeberger et al., 2020). The AS analysis suggests that the *Rbm42* is important for the regulation of gene splicing in neurodevelopment.

## Discussion

NDDs are a group of genetically heterogeneous disorders most of which lack distinctive clinical phenotypes for a prompt diagnosis. Comprehensive genetic testing is useful to identify the underlying etiology in NDD patients and facilitate the discovery of novel disease genes (Savatt and Myers, 2021). In this work, exome analysis coupled with model organism studies linked two variants in the *RBM42* gene, c.304C>T (p.R102*) and c.1312G>A (p.A438T), to a syndromic recessive NDD characterized by severe CNS defects, dysmorphism, global development delay, and congenital cardiac defects. *RBM42* interacts with the gene product of *HNRNPK*, the causative gene of AKS, which has a plethora of overlapping neurological and physical symptoms as seen in the index patient (Table 1). While the p.R102* variant is a null allele, the p.A438T variant in *RBM42* partly disrupts its interaction with hnRNP K (Fig. 3G and 3H), which may lead to the dysfunction of their common downstream effectors and cause similar symptoms in patients. Despite their phenotypic similarities (Table 1), the index patient does not have several symptoms found in some of the AKS patients such as hydronephrosis, scoliosis, and pes planus, which may be explained by unknown genetic or environmental modifiers causing phenotypic variability. Note that *RBM42* identified in this study is another RBM gene associated with a human monogenic disease. Patients affected by *RBM42*, *RBM8A,* and *RBM10* pathogenic variants all have neurological deficits, but they present with distinct CNS phenotypes. For instance, patients with *RBM8A* and *RBM10* pathogenic variants have cerebellar vermis hypoplasia (Skórka et al., 2005; Kaeppler et al., 2018), which is not seen in the index patient. Such phenotypic differences may be caused by distinct roles of RBM proteins in the regulation of RNA processing. RBM8A participates in non-sense mediated mRNA decay, RBM10 regulates gene transcription, and *RBM42* interacts with hnRNP K to regulate AS (Fukuda et al., 2009; Charenton et al., 2019; McSweeney et al., 2020; Inoue, 2021). Therefore, with a fine-tuned spatial and temporal activity, these RBM proteins may function in different RNA-binding protein complexes required for normal embryonic development.

The fungus and the mouse mutant model in this study recapitulated the development defects seen in the patient (Figs. 4 and 5). However, additional patients were not found to date with biallelic pathogenic variants in the *RBM42* gene despite an extensive search in both public databases and major clinical laboratories around the world. Individuals carrying homozygous null alleles in *RBM42* may not survive and those with homozygous hypomorph missense variants may be asymptomatic, which could have made the search difficult. However, it is critical to identify additional patients with *RBM42* loss-of-function variants to substantiate their causality in the context of CNS and other phenotypes. Given its indispensable role for normal embryonic development, the identification of such patients is more likely to be fruitful in those who carry a truncating variant and a missense variant harboring residual *RBM42* function.

To investigate how *RBM42* may regulate embryonic development, transcriptomic profiles produced by RNA-seq were compared between mouse models with the patient’s pathogenic variants. The enriched dysregulated pathways in the mutant mice were mostly involved in neurological and myocardial function, coinciding with the neurological and cardiac defects seen in the patient (Figs. 6C, 6D and S4–6). Differentially expressed or spliced genes in the compound heterozygous mutant animals include ortholougus genes causing human neurological disorders such as *Abca7, Zfp365, Kmt2c, Madd* and *Tcf4* (Figs. 6E and 7D). Further research will be focused on the study how *RBM42* fine-tunes these genes in embryonic development.

Overall, we herein identified the pathogenic variants in the *RBM42* gene as the underlying genetic etiology associated with a previously unrecognized syndromic NDD. Results from the clinical evaluation, genetic analysis, cellular functional assay, and mutant model systems support that *RBM42* has an essential role linking global AS to proper cellular growth required for normal embryonic development.

## Materials and methods

### Ethics statement

This study was conducted in accordance with the Declaration of Helsinki and approved by the Ethics Review Committee of the International Peace Maternity and Child Health Hospital (IPMCH), Shanghai Jiao Tong University School of Medicine (GKLW 2020-42). Informed consents were obtained from all participants or their legal guardians, and peripheral blood samples were collected for further analysis.

### Whole-exome sequencing and data analysis

Genomic DNA was extracted from peripheral blood samples using the blood genomic extraction kit (Qiagen, Germany) and subjected to targeted next-generation sequencing. The coding exons and flanking sequences of over 20,000 genes were captured for paired-end sequencing (2× 100 bp) on Illumina HiSeq2500 (Illumina, USA). The average sequencing depth of the target region was higher than 170×, with over 95% of bases covered by at least 30×. An in-house genome analysis pipeline composed of BWA 0.7.12 (Li and Durbin, 2009), GATK 3.5 (McKenna et al., 2010; DePristo et al., 2011), snpEff 4.0 (Cingolani et al., 2012), BED Tools 2.25.0 (Quinlan, 2014), ANNOVA (Yang and Wang, 2015) were used to process demultiplexed fastq data. BAM files were generated to visualize read pairs and variant calling in IGV (Broad Institute, USA). Candidate variants were filtered based on the population frequency of 2%, predicated variant pathogenicity, inheritance mode, and protein interaction network.

### Variant confirmation by Sanger sequencing

The variants in the candidate disease genes identified by WES were confirmed by Sanger sequencing. The primers for the c.304C>T variant (*RBM42*-C304-F: 5ʹ-cctgagatagccagccacat-3ʹ and *RBM42*-C304-R: 5ʹ-actctttacttaccagggcct-3ʹ) and c.1312G>A variant (*RBM42*-C1312-F: 5ʹ-agacaaggtagacactgggc-3ʹ and *RBM42*-C1312-R: 5ʹ-gagacagagggttcaaggca-3ʹ) were designed by Primer 3 (Untergasser et al., 2012). PCR was performed using 25 μL 2×Taq MasterMix (CWBIO, China), 2 μL primer mix (10 μmol/L), 100 ng genomic DNA template per sample, and RNase-free water up to 50 μL. The PCR program was 95°C for 5 min, 35 cycles for (95°C for 30 s, 59°C for 30 s, 72°C for 30 s), 72°C for 5 min, and 4°C hold.

### RT-PCR and qRT-PCR

Total RNA was extracted using TRIzol (Invitrogen, USA), and cDNA was synthesized using the PrimeScript RT reagent Kit with gDNA Eraser (TaKaRa, Japan). The cDNA products were subjected to RT-PCR analysis and the products were separated on 2% agarose gel. The qPCR was performed using TB Green Premix Ex Taq (Tli RNaseH Plus) (TaKaRa, Japan) on ABI 7500 (Applied Biosystems, USA) according to the manufacturer’s protocol. Primers are listed in Table S1.

### Establishment of lymphoblastoid cell lines by immortalizing lymphocytes

Peripheral blood was collected into a sterile heparin tube, diluted with serum-free RPMI 1640 (1:1), and slowly dropped onto the surface of lymphocyte separation solution. The samples were centrifuged at 2,500 rpm for 10 min to separate lymphocytes, and washed the cell precipitate twice with RPMI 1640. Next, 100 μL (200 μg/mL) cyclosporine A, 1.5 mL EB virus liquid, and 2 mL complete medium (25% Fetal Bovine Serum + 75% RPMI 1640, Gibco, USA) were added to gently pipette the cells into a single lymphocyte suspension. The lymphocyte suspension was transferred to a T25 flask for open culture at 37°C with 5% CO_2_. Flow cytometric identification, STR identification, mycoplasma detection, and sterility tests were performed to confirm the successful establishment of LCLs.

### Cell culture and transfection

The FLAG-*RBM42*-WT plasmid was obtained from Vectorbuilder (China). The FLAG-*RBM42*-R102* and FLAG-*RBM42*-A438T constructs were generated by Sangon Biotech (China). The sequences of all plasmids were confirmed by Sanger sequencing. 293T and HeLa cells were cultured in DMEM/HIGH GLUCOSE (Gibco, USA) supplemented with 10% (*v*/*v*) Fetal Bovine Serum (Gibco, USA) at 37°C in a humidified atmosphere with 5% CO_2_. For transient transfection, 293T cells were seeded at a density of 6 × 10^5^ cells per well and grown to 70%–80% confluence. The cells were then transfected using the Lipofectamine 3000 (ThermoFisher, USA) following the manufacturer’s instructions. After an additional 48 h of incubation, the cells were harvested.

### Immunoblotting

The monoclonal anti-FLAG M2, the polyclonal anti-HA, the anti-Mouse IgG (whole molecule) peroxidase antibody and the anti-Rabbit IgG (whole molecule) peroxidase antibody were from Sigma (USA). The polyclonal anti-*RBM42* was from Abcam (UK). The monoclonal hnRNP K antibody was from Santa Cruz (USA). The HRP-conjugated Beta Actin monoclonal antibody was from Proteintech (USA).

The cells were washed with ice-cold PBS and then harvested in lysis buffer (150 mmol/L NaCl, 20 mmol/L Tris, 1% Triton X-100) supplemented with protease inhibitor cocktail (MedChemExpress, USA). The lysates were incubated for 30 min in lysis buffer at 4°C and then centrifuged for 15 min at 16,000 ×*g*. The protein concentration was measured using BCA Protein Assay Kit (TaKaRa, Japan). The cell lysates were denatured using 5× SDS sample buffer and analyzed by immunoblotting with specific antibodies. Images were analyzed by the ImageJ software (1.8.0).

### Immunoprecipitation

293T cells were transiently transfected with FLAG-tagged *RBM42*-WT, R102*, A438T or control vector as described above for 48 h. The cells were then washed with ice-cold PBS and harvested in 500 μL of NETN lysis buffer (250 mmol/L NaCl, 5 mmol/L EDTA, 50 mmol/L Tris-HCl,0.5% NP40) with protease inhibitor tablets (Roche, Switzerland). After 10 min of incubation in lysis buffer at 4°C, the lysates were then centrifuged for 5 min at 16,000 ×*g* at 4°C. One thousand microgram of protein was used for immunoprecipitation. One microgram of FLAG antibody was added to the supernatant. Incubation at 4°C overnight, 20 μL of Protein A/G Plus-Agarose (Santa Cruz, USA) was added, followed by an hour incubation at 4°C. Samples were then centrifuged 5 min at 1000 ×*g* and washed three times with 1 mL of lysis buffer. Immunoprecipitated proteins were eluted by addition of 30 μL of 1× SDS sample buffer, followed by 10 min incubation at 100°C. Initial lysates and immunoprecipitated proteins were analyzed by immunoblotting with specific antibodies.

### Immunofluorescence

The polyclonal anti-*RBM42*, the donkey anti-Mouse IgG (H+L) Highly Cross-Adsorbed Secondary Antibody (Alexa Fluor 488), and the donkey anti-Rabbit IgG (H+L) Highly Cross-Adsorbed Secondary Antibody (Alexa Fluor 555) were from ThermoFisher (USA).HeLa cells were plated in 24-well plates containing coverslip at a density of 3 × 10^4^ cells per well. To detect the overexpression of *RBM42*, the cells were transiently transfected with 1 μg DNA of FLAG-tagged *RBM42*-WT, R102* or A438T on the following day. Endogenous gene expression analysis was performed using untransfected HeLa cells. After 2 days of transfection, the cells were fixed with 4% (*v*/*v*) paraformaldehyde for 15 min and permeabilized with 0.5% (*v*/*v*) Triton X-100 for 30 min at room temperature. The specimens were then blocked with 5% BSA and incubated with specific antibodies overnight at 4°C. The next day, the coverslips were washed with PBS and incubated with the appropriate secondary antibodies for an hour at room temperature. After washing with PBS, the coverslips were mounted using VECTASHIELD (USA) antifade reagent with DAPI (VECTOR, USA) and observed by confocal microscopy.

### Fungal strains, culture conditions, and construction of complementation strains

*Fusarium graminearum* strain PH-1 (NRRL 31084) was used to construct FgRbp1 gene-deletion mutants. Fungal strains were cultured at 25°C on potato dextrose agar (PDA) for mycelial growth tests. Putative gene-deletion mutants were obtained in previous study (Wang et al., 2021). The coding sequence of FLAG-*RBM42*-WT, FLAG-*RBM42*-R102* and FLAG-*RBM42*-A438T were cloned into fungal expression vector pYF11 to generate *RBM42*^WT^, *RBM42*^R102*^ and *RBM42*^A438T^ complemented plasmids, respectively. The construct expressing human WT, R102* and A438T mutant *RBM42* were independently transformed into ΔFgRbp1. The transformation of *F*. *graminearum* was carried out via polyethylene glycol-mediated protoplast transformation method (Proctor et al., 1995). The resulting strains ΔFgRbp1::*RBM42*^WT^, ΔFgRbp1::*RBM42*^R102*^, and ΔFgRbp1::*RBM42*^A438T^, along with the control strains ΔFgRbp1 and PH-1, were cultured on PDA at 25°C for 3 days to assess mycelial growth and colony phenotype.

### Mice

Mouse models with *Rbm42* variant, Q94* and A436T, were generated through cytoplasm injection of CRISPR/Cas9 genome editing tools, concluding Cas9 mRNA (100 ng/μL), single-guide RNA (100 ng/μL) and single-stranded oligodeoxynucleotide (100 ng/μL) as homology-directed repair template. Single-guide RNAs were synthesized by *in vitro* transcription using the MEGAshortscript Kit (ThermoFisher, USA). Cas9 mRNA was prepared by *in vitro* transcription using the mMESSAGE mMACHINET7 ULTRA Transcription Kit (ThermoFisher, USA). Both Cas9 mRNA and single-guide RNA were purified with MEGA clear kit (ThermoFisher, USA) and eluted with RNase-free water. The primers for verifying Q94* (c.280C>T) variant (*Rbm42*-P94-F: 5ʹ-gccctggttagtccttggat-3ʹ and *Rbm42*-P94-R: 5ʹ-ttaccaccctgcctgaaaca-3ʹ) and A436T (c.1306_1308delinsACA) variant (*Rbm42*-P436-F: 5ʹ-actgaaggttctggggatgg-3ʹ and *Rbm42*-P436-R: 5ʹ-agggctcactcaccattcat-3ʹ) were designed by primer 3 online (Untergasser et al., 2012). Embryos with double variant were generated by *in vitro* fertilization (IVF) with MII oocytes from female A436T/WT and sperm from male Q94*/WT. 16–20 embryos were transferred into oviducts of each pseudopregnant ICR female. Three, three, and six surrogate mice were harvest at E9.5, E13.5 and D0, respectively. All animal procedures were performed following the ethical guidelines and regulations of Institute of Biochemistry and Cell Biology, Shanghai Institutes for Biological Sciences, Chinese Academy of Sciences.

### RNA-seq and data analysis

Over 1 μg total RNA per sample was used for the RNA-seq. mRNA was purified by Poly-T oligo-attached magnetic beads and fragmented for cDNA synthesis. Libraries were generated using the NEBNext Ultra RNA Library Pre Kit (NEB, USA) following the manufacturer’s instructions. Sequencing was performed on the Illumina NovaSeq 6000 platform (Illumina, USA). HISAT2 (v2.0.5), featureCounts (1.5.0-p3), DESeq2 (1.20.0), clusterProfiler (3.4.4), GSEA, diamond (0.9.13) and rMATS (3.2.5) were used to sequencing data analysis.

### Differential expression analysis

Differential expression analysis of two groups was performed using the DESeq2 R package (1.20.0). DESeq2 provide statistical routines to determine differential expression in digital gene expression data using a model based on the negative binomial distribution. Genes with an *P*-value < 0.05 and fold change ≥ 1.5 were considered differentially expressed.

### Enrichment analysis of differentially expressed genes

GO enrichment analysis of DEGs was implemented by the clusterProfiler R package, in which gene length bias was corrected. GO terms with *P* value < 0.05 were considered significantly enriched by differential expressed genes. KEGG is a database resource for understanding high-level functions and utilities of the biological system, such as the cell, the organism and the ecosystem, from molecular-level information, especially large-scale molecular datasets generated by genome sequencing and other high-through put experimental technologies. We used clusterProfiler R package to test the statistical enrichment of differential expression genes in KEGG pathways.

### Alternative splicing analysis

Alternative splicing is an important mechanism for regulate the expression of genes and the variable of protein. rMATS (4.0.2) software was used to analysis the AS event. The resulting *P* values were adjusted using the Benjamini and Hochberg’s approach for controlling the FDR. Alternative splicing events with FDR < 0.05 were considered significantly different.

## Supplementary Material

pwad034_suppl_Supplementary_Materials

## Data Availability

The raw data supporting the conclusions of this article will be made available by the authors, without undue reservation, to any qualified researcher.

## Abbreviations

A3SS: alternative 3ʹ splicing site
A5SS: alternative 5ʹ splice site
AKS: Au-Kline syndrome
AS: alternative splicing
CNS: central nervous system
Ctrl: control
DEGs: differentially expressed genes
FDR: false discovery rate
GO: Gene Ontology
hnRNP: heterogeneous nuclear ribonucleoprotein
KEGG: Kyoto encyclopedia of genes and genomes
LCLs: lymphoblastoid cell lines
MXE: mutually exclusive exons
NA: not applicable
NDDs: neurodevelopmental disorders
PDA: potato dextrose agar
RBM: RNA-binding motif
RBPs: RNA-binding proteins
RI: retained intron
RRM: RNA recognition motif
SE: skipped exon
WES: whole-exome sequencing

## Code availability

No code or model was generated in this study.
