# Supplementary material for: Biallelic variants in RBM42 cause a multisystem disorder with neurological, facial, cardiac, and musculoskeletal involvement
Source: Protein Cell. 2023 Jun 9;15(1):52–68. doi: 10.1093/procel/pwad034 (PMC10762670; doi:10.1093/procel/pwad034)
Supplement: pwad034_suppl_Supplementary_Materials [file pwad034_suppl_supplementary_materials.pdf]

Supplementary Material

Fig S1 *De novo WAS* variant of the proband. Sanger sequencing confirmed the *de novo* heterozygous splicing variant of the proband (II-2) in *WAS*.

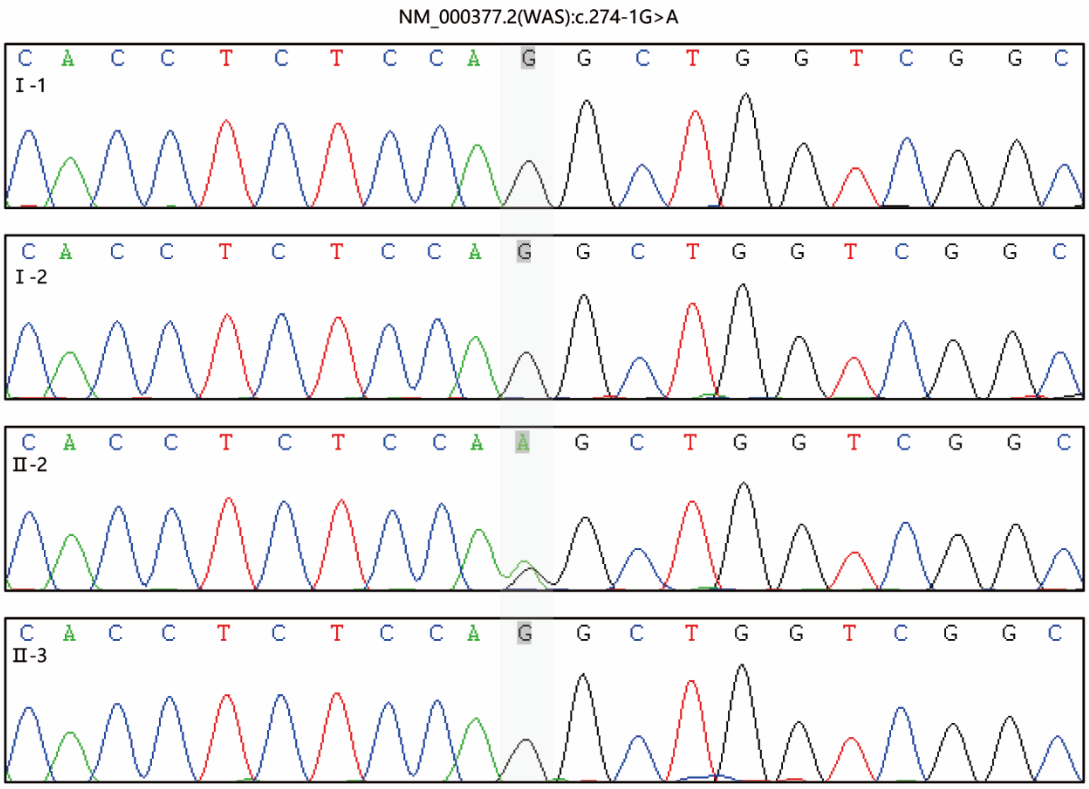

Fig S2. RBM42 orthologs are conserved in fungi, metazoa and plants, but not in Saccharomyces species. The phylogenetic tree was constructed based on the amino acid sequences of RBM42 and its orthologs in other organisms with Mega 11.0 using the neighbor-joining method. The analyzed organisms include Arabidopsis thaliana (NP_187100.1), Aspergillus nidulans (XP_661557.1), Botrytis cinerea (XP_001558675.1), Caenorhabditis elegans (NP_498090.1), Cryptococcus neoformans (XP_012050974.1), Drosophila melanogaster (NP_649552.1), Homo sapiens (NP_077297.2), Magnaporthe oryzae (XP_003714189.1), Mus musculus (NP_598454.2), Neurospora crassa (XP_964958.2), Schizosaccharomyces pombe (NP_594828.1), and Ustilago maydis (XP_011387189.1).

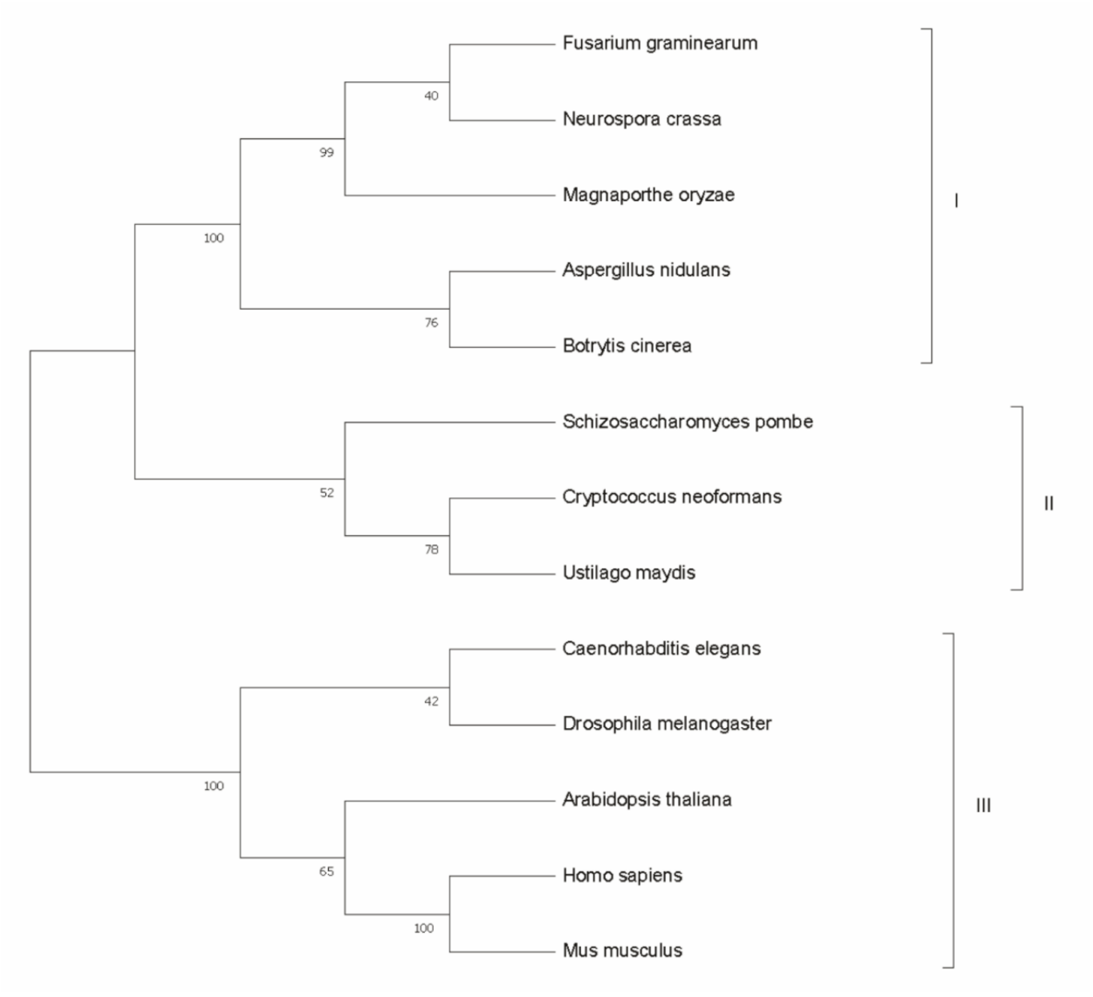

Fig S3 Amino acid alignment of RBM42 orthologs reveals that the RRM domain of FgRbp1 is conserved among different species. Hs, Homo sapiens; Mm, Mus musculus; At, Arabidopsis thaliana; An, Aspergillus nidulans; Bc, Botrytis cinerea; Ce, Caenorhabditis elegans; Cn, Cryptococcus neoformans; Dm, Drosophila melanogaster; Mo, Magnaporthe oryzae; Nc, Neurospora crassa; Sp, Schizosaccharomyces pombe; and Um, Ustilago maydis. The black box indicates the RRM domain of RBM42. The red inverted triangle indicates the mutation position of c.1312G>A (p.A438T) in human *RBM42*.

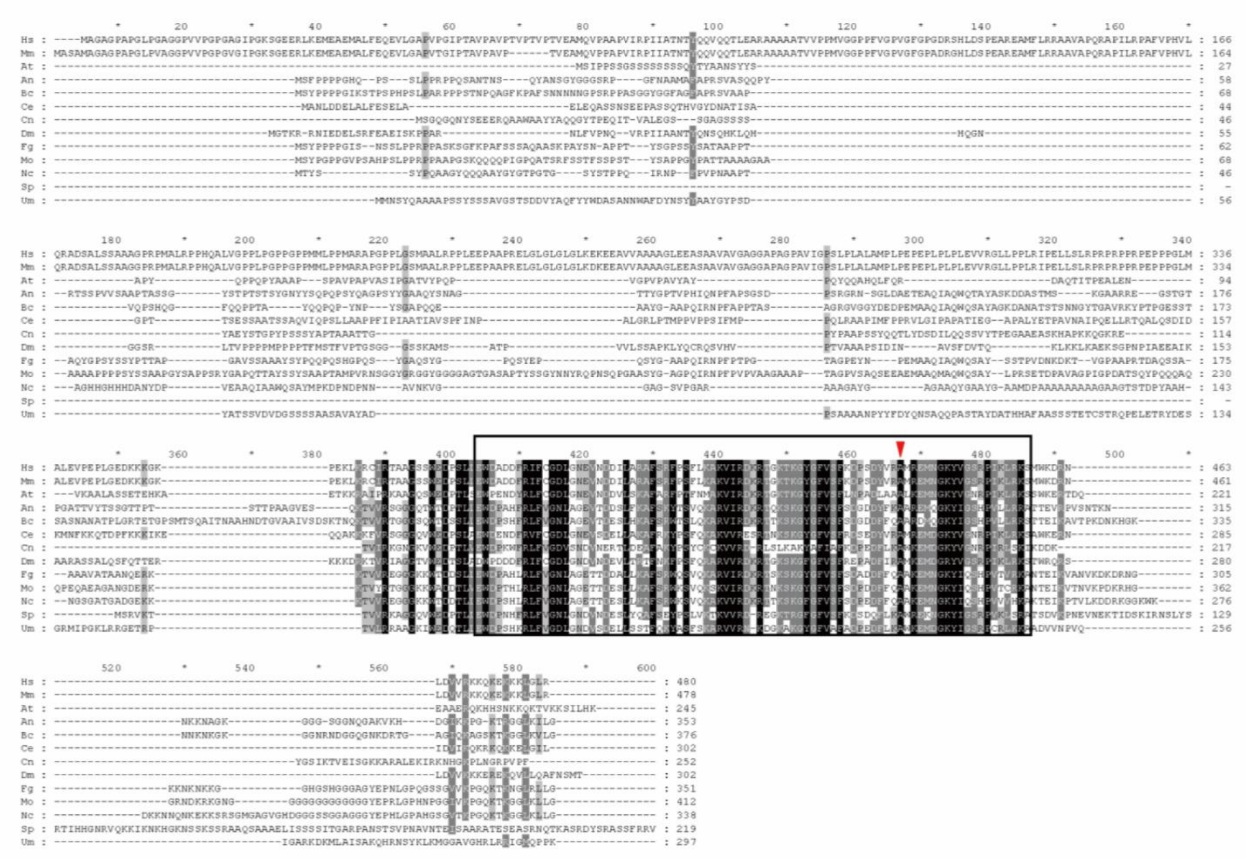

Fig S4 Top 30 terms in biological process of Gene Ontology functional enrichment of upregulated (A) and downregulated (B) differentially expressed genes in M1M2 E9.5 mouse embryo compared to WT.

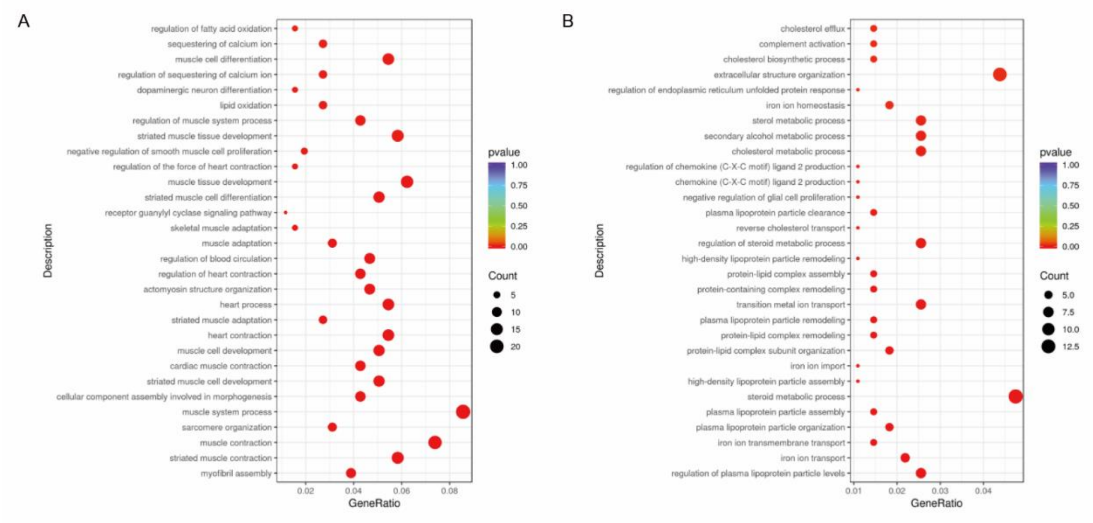

Fig S5 Functional enrichment analysis of differentially expressed genes in M1M2 E9.5 mouse embryo compared to M1. (A) Top 10 enriched terms in Kyoto Encyclopedia of Genes and Genomes. (B) The dotplot for Top 10 molecular function, 10 cellular component and 10 biological process terms enriched in Gene Ontology.

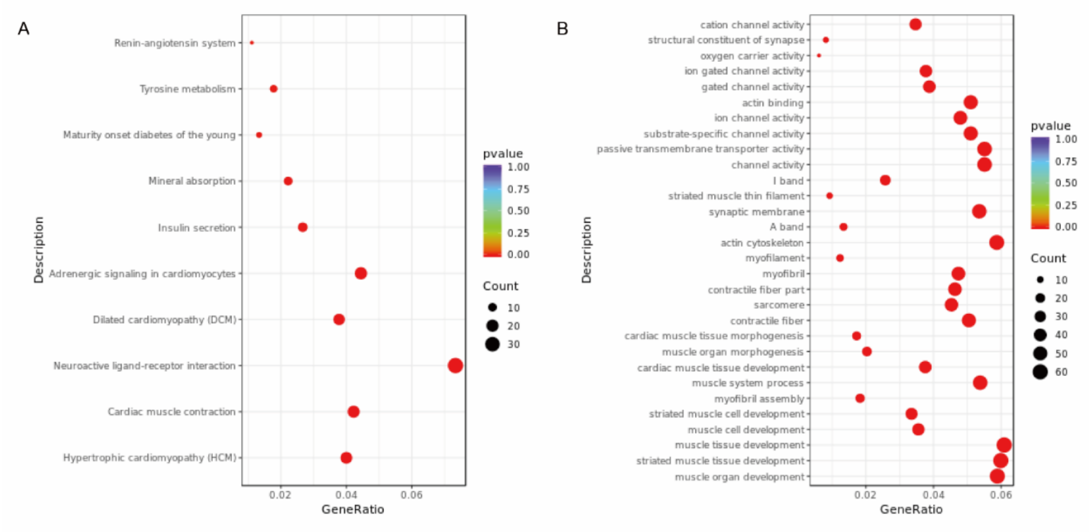

Fig S6 Functional enrichment analysis of differentially expressed genes in M1M2 E9.5 mouse embryo compared to M2. (A) Top 10 enriched terms in Kyoto Encyclopedia of Genes and Genomes. (B) The dot plot for Top 10 molecular function, 10 cellular component and 10 biological process terms enriched in Gene Ontology.

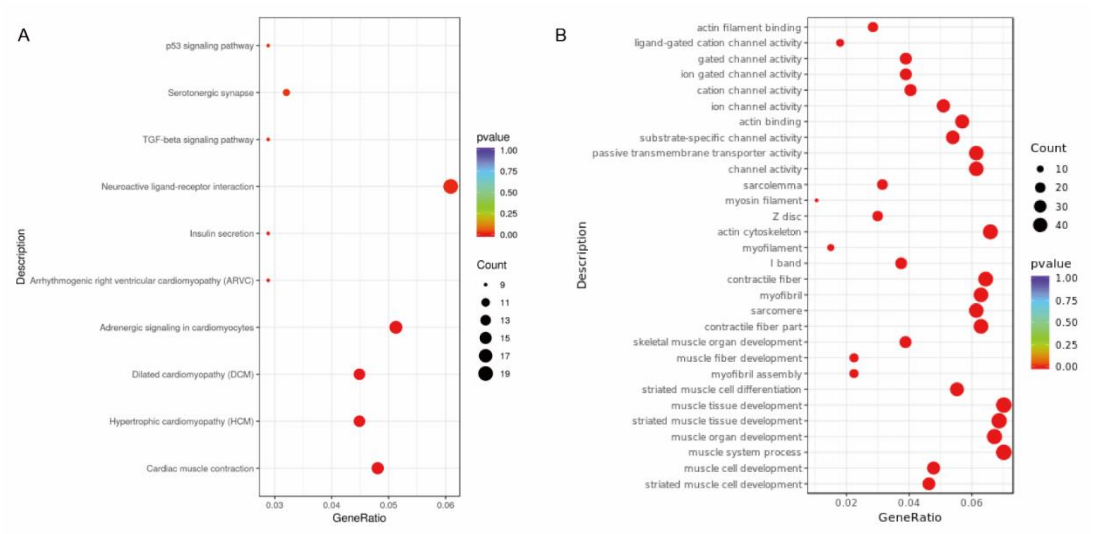

Fig S7 Alternative splicing analysis in M1M2 E9.5 mouse embryo compared to WT. (A) Kyoto Encyclopedia of Genes and Genomes pathway analysis of skipped exon genes. (B-C) Gene Ontology analysis showed the enrichment of genes involved in alternative splicing, including cellular components (B) and molecular function (C).

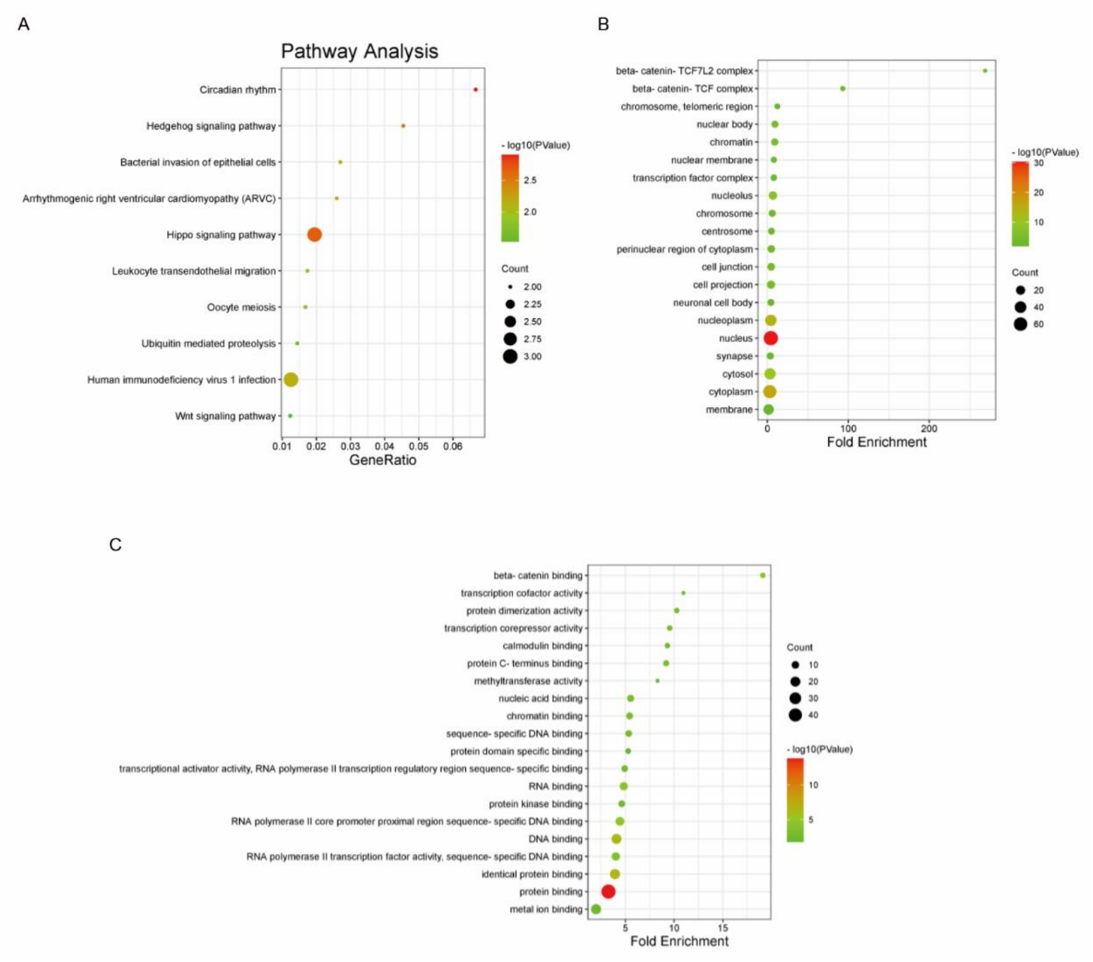

Table S1 Primers used in quantitative real-time reverse transcription-polymerase chain reaction (qRT-PCR) and reverse transcription-polymerase chain reaction (RT-PCR).

Gene	Forward (5'-3')	Reverse (5'-3')
RBM42	GAGAAGCCATGTTCCTGCGG	GCAGAGGAGAGAGCGGAATC
ACTIN	CCTGGCACCCAGCACAAT	GGGCCGGACTCGTCATAC
Rbm42	TAGAAGCGATGCAGGTCCC	CTGCTCGCCTCAGGAACAT
Actin	ATATCGCTGCGCTGGTCGTC	AGGATGGCGTGAGGGAGAGC
Abca7	CCGAGGCCTAAAGACCAAGA	CAGCCCTTGTTGTTGAACCA
Aga	GTGTGATGGGACTGTAGGCT	AAAGCGTGTGTGTGGTATGC
Ak1	CAATGGCTTCCTGATCGACG	GCCACGCTTGTCATAGAAGG
Casq1	GCGAGTTTTCTGCAGACACT	GATGGAACTCTTCAGCTGCG
Chrd	GTTCTATTGGCTGGGCTTGG	ACTCTTGGTGGTGATGAGCA
Dcn	CAACAAACTCCTCAGGGTGC	AGGGTTGCCGTAAAGACTCA
Ddit4l	CGGGCAGTTTGAGCAGTAAG	GGGACCAAGACCTTAGAGCA
Dusp27	AACGAAGAACTCAAGCCTCG	CAGCTATGAAGACGTTGGGC
Gpcpd1	GAACTCAACAACGACTGCCA	GATACACCACGGGCACAAAG
Gpr50	ATGGTCATCACAGTCGTCGT	GCAGTAACGGTTGATGGCAA
Kntc1	ACTTTTGCGAGGGTATGGGA	TCGAGCCCAGATGATAACCC
Lmod1	TCCCCTGAAGAGATGGAGGA	CATCGCTGTCCTTCTCCTCT
Ndufaf6	CTGGATGACAAAGCGTACCG	ACCTGTCTCCTGCTACTGTG
Zfp365	TGCTGCAACGGGTAGAATTG	CTCGCTCATCCTTGGCTTTC
Hnrnpa2b1	GTCTTTCTCATCTCGCTCGG	AGTCTGTGAGCTTTCCCCAT
Kmt2c	TGGCCGTAATCCTCTCATGG	CTGGTGCTATTCAAGGTGTGA
Madd	TCTGTCATCGGTGTGAGTCC	AGGTCACTGTCAGCTCCAAG
Tcf4	GCGTGTCTTCTGGTAGCTCT	GTCAGGTCCTCATCATCGTTAT
Tcf7l2	AATCCACCTCCGCACTTACC	TCCTGTCGTGATTGGGTACA
Hprt	TGATGAACCAGGTTATGACC	GTTGAGAGATCATCTCCACC

Table S2 Quantification of differentially expressed genes expression involved in neurological function in mutant and control E9.5 mouse embryos RNA-seq.

Genes involved in neurological function												
	M1M2 vs WT		M1M2 vs M1		M1M2 vs M2							
Gene symble	log_2Fold Change	p value	log_2FoldChange	p value	log_2Fold Change	p value	Gene full name	KEGG annotation	GO category	GO annotation	MIM number	Gene-Phenotype Relationships
Gpr50	-0.90	4.58E-04	-0.85	4.84E-05	-0.83	7.43E-05	G-protein-coupled receptor 50	Neuroactive ligand-receptor interaction	/	/	300207	
Chrd	-1.01	1.34E-02	-0.94	2.86E-03	-1.02	1.63E-04	chordin	TGF-beta signaling pathway	BP<br>BP<br>BP<br>BP<br>BP<br>BP<br>BP	spinal cord development<br>forebrain development<br>regionalization<br>pattern specification process<br>negative regulation of nervous system development<br>spinal cord dorsal/ventral patterning<br>spinal cord patterning	603475	
Abca7	-1.01	1.21E-05	-1.20	1.78E-08	-1.17	1.87E-11	ATP-binding cassette, sub-family A (ABC1), member 7	ABC transporters	BP<br>BP<br>BP<br>BP<br>CC<br>CC	memory<br>learning or memory<br>cognition<br>associative learning<br>cell projection membrane<br>glial cell projection	605414	Alzheimer disease 9, susceptibility to
Ndufaf6	1.03	1.58E-14	0.92	2.05E-19	0.99	1.26E-25	NADH:ubiquinone oxidoreductase complex assembly factor 6	/	CC<br>CC	organelle inner membrane<br>mitochondrial inner membrane	612392	Mitochondrial complex I deficiency, nuclear type 17
Ddit4l	0.96	1.81E-05	0.65	7.79E-04	0.78	7.38E-06	DNA-damage-inducible transcript 4-like	/			607730	
Aga	0.85	1.64E-06	0.84	2.61E-07	0.83	1.79E-09	aspartylglucosaminidase	/			613228	Aspartylglucosaminuria
Zfp365	1.07	2.49E-07	1.20	2.52E-12	1.34	2.51E-16	zinc finger protein 365	/	BP<br>BP<br>BP	synapse organization<br>hindbrain development<br>dendrite morphogenesis	607818	Nephrolithiasis, uric acid, susceptibility to

		BP	dendrite development
		BP	negative regulation of neurogenesis
		BP	cerebellum development
		BP	negative regulation of nervous system development
		BP	metencephalon development

KEGG: Kyoto Encyclopedia of Genes and Genomes
GO: Gene Ontology
BP: Biological Process
CC: Cellular Component
MIM: Mendelian Inheritance in Man

Table S3 Quantification of differentially expressed genes expression involved in myocardiac function in mutant and control E9.5 mouse embryos RNA-seq.

Genes in myocardiac function												
	M1M2 vs WT		M1M2 vs M1		M1M2 vs M2							
Gene symble	log_2FoldChange	p value	log_2FoldChange	p value	log_2FoldChange	p value	Gene full name	KEGG annotation	GO category	GO annotation	MIM number	Gene-Phenotype Relationships
Dcn	-1.36	8.71E-09	-0.94	1.16E-04	-1.09	2.45E-10	decorin	/	BP<br>BP<br>BP<br>BP<br>BP	muscle organ development<br>striated muscle tissue development<br>muscle cell development<br>skeletal muscle organ development<br>muscle tissue development	125255	Corneal dystrophy, congenital stromal
Kntc1	-1.05	3.16E-15	-0.90	3.26E-16	-0.82	1.24E-13	kinetochore associated 1	/	CC	actin cytoskeleton	607363	
Gpcpd1	-0.93	1.76E-09	-0.79	8.69E-08	-0.65	2.07E-07	glycerophosphocholine phosphodiesterase 1	/	BP<br>BP<br>BP<br>BP	muscle organ development<br>striated muscle tissue development<br>muscle tissue development<br>skeletal muscle organ development	614124	
Lmod1	0.91	1.19E-03	0.76	1.54E-03	0.69	3.43E-03	leiomodin 1 (smooth muscle)	/	BP<br>CC<br>CC<br>CC<br>CC<br>CC<br>MF<br>MF	muscle system process<br>sarcomere<br>contractile fiber part<br>contractile fiber<br>myofibril<br>actin cytoskeleton<br>tropomyosin binding<br>actin binding	602715	Megacystis-microcolon-intestinal hypoperistalsis syndrome 3
Ak1	0.99	1.21E-09	0.90	4.71E-14	0.87	6.00E-18	adenylate kinase 1	/	CC<br>CC<br>CC<br>CC	sarcomere<br>contractile fiber part<br>contractile fiber<br>myofibril	103000	Hemolytic anemia due to adenylate kinase deficiency
Casq1	0.75	1.83E-02	0.74	1.85E-07	0.71	5.89E-06	calsequestrin 1	/	BP<br>BP<br>BP<br>BP<br>BP<br>BP<br>BP<br>BP<br>BP<br>CC	striated muscle contraction<br>myofibril assembly<br>sarcomere organization<br>striated muscle cell development<br>muscle system process<br>muscle organ development<br>striated muscle tissue development<br>muscle tissue development<br>muscle cell development<br>sarcomere	114250	Myopathy, vacuolar, with CASQ1 aggregates

									CC	contractile fiber part	
									CC	contractile fiber	
									CC	myofibril	
									CC	Z disc	
									CC	I band	
									CC	sarcoplasmic reticulum membrane	
Dusp27	0.90	8.49E-06	0.87	3.97E-08	0.76	4.17E-06	dual specificity phosphatase 27 (putative)	/	CC	sarcomere	618574
									CC	contractile fiber part	
									CC	contractile fiber	
									CC	myofibril	

KEGG: Kyoto Encyclopedia of Genes and Genomes
GO: Gene Ontology
BP: Biological Process
CC: Cellular Component
MF: Molecular Function
MIM: Mendelian Inheritance in Man

Table S4 *Rbm42*-regulated alternative splicing events identified by RNA-Seq in E9.5 mouse embryos.

SE

GeneID	geneSymbol	chr	strand	exonStart	exonEnd	upstreamES	upstreamEE	downstreamES	downstreamEE	P-value	FDR	IncLevelDiffer
ENSMUSG00000029090	Adgra3	5	-	50058133	50058343	50050868	50050960	50058551	50058645	7.42E-07	0.0013	0.720
ENSMUSG00000058318	Phf21a	2	+	92186550	92186609	92185458	92185588	92214048	92214089	4.42E-05	0.0269	0.611
ENSMUSG00000044501	Zfp758	17	+	22372035	22372088	22361521	22361707	22373642	22373769	1.19E-05	0.0111	0.574
ENSMUSG00000034157	Cipc	12	+	86959727	86959828	86952743	86953004	86960271	86960391	4.61E-05	0.0269	0.569
ENSMUSG00000085614	1700123M08Rik	4	+	11976443	11976587	11966573	11966683	11993864	11993999	3.48E-07	0.0007	0.549
ENSMUSG00000087138	Gm15545	7	+	44991376	44991481	44986899	44987370	44993982	44994597	3.12E-05	0.0206	0.542
ENSMUSG00000044501	Zfp758	17	+	22371990	22372088	22361452	22361707	22373642	22373769	3.73E-06	0.0045	0.536
ENSMUSG00000061689	Dlgap4	2	+	156748022	156748102	156745834	156746196	156748943	156749356	3.04E-05	0.0205	0.517
ENSMUSG00000024743	Syt7	19	+	10422784	10423012	10421756	10421888	10426298	10426395	1.42E-05	0.0120	0.509
ENSMUSG00000037994	Slc9b2	3	+	135315880	135315950	135307730	135307781	135317053	135317193	1.24E-05	0.0111	0.504
ENSMUSG00000021495	Fam193b	13	-	55564519	55564580	55555982	55556225	55569704	55571120	2.32E-06	0.0034	0.486
ENSMUSG00000020397	Med7	11	+	46439805	46439824	46436970	46437191	46440562	46441100	7.40E-05	0.0362	0.480
ENSMUSG00000065952	C330021F23Rik	8	+	3573120	3573221	3567994	3568096	3577840	3578045	6.09E-09	0.0000	0.413
ENSMUSG00000085396	Firre	X	-	50560808	50561006	50558230	50558384	50562679	50562954	2.92E-05	0.0201	0.387
ENSMUSG00000049107	Ntf3	6	-	126164729	126164840	126101411	126102523	126166539	126166715	1.71E-07	0.0004	0.382
ENSMUSG00000058486	Wdr91	6	-	34909713	34909827	34909460	34909540	34910696	34910842	5.22E-15	0.0000	0.344
ENSMUSG00000022607	Ptk2	15	-	73392343	73392447	73391758	73391880	73394647	73394744	1.08E-07	0.0003	0.321
ENSMUSG00000026955	Sapcd2	2	+	25373415	25373517	25372320	25372998	25374951	25375064	8.64E-14	0.0000	0.263
ENSMUSG00000020271	Fbxw11	11	+	32708187	32708250	32680200	32680302	32711771	32711997	3.42E-06	0.0045	0.259
ENSMUSG00000056211	R3hdm1	1	+	128190637	128190739	128186757	128186909	128193459	128193756	1.33E-09	0.0000	0.234
ENSMUSG00000031816	Mthfsd	8	-	121107521	121107642	121106869	121106973	121108287	121108392	2.79E-05	0.0197	0.220
ENSMUSG00000067367	Lyar	5	+	38223133	38223227	38220519	38220635	38224626	38224797	1.59E-14	0.0000	0.214
ENSMUSG00000023007	Prpf40b	15	+	99305165	99305193	99304424	99304490	99305407	99305433	2.09E-07	0.0005	0.197
ENSMUSG00000019478	Rab4a	8	+	123828995	123829058	123823813	123823894	123830270	123830387	6.30E-05	0.0323	0.192
ENSMUSG00000032178	Ilf3	9	+	21388111	21388150	21387699	21387797	21388508	21388753	8.21E-05	0.0377	0.188

ENSMUSG00000041225	Arhgap12	18	-	6057516	6057591	6052861	6052923	6061846	6061972	4.14E-06	0.0048	0.181
ENSMUSG00000058486	Wdr91	6	-	34909676	34909827	34909360	34909540	34910696	34910836	5.78E-05	0.0313	0.178
ENSMUSG00000030609	Aen	7	+	78902229	78902830	78895926	78896014	78905842	78906043	7.20E-06	0.0077	0.163
ENSMUSG00000039298	Cdk5rap2	4	-	70246947	70246974	70245380	70245497	70250352	70250472	1.88E-05	0.0146	0.162
ENSMUSG00000030609	Aen	7	+	78902343	78902830	78895933	78896014	78905842	78906043	1.02E-05	0.0103	0.154
ENSMUSG00000004980	Hnrnpa2b1	6	-	51467390	51467426	51467184	51467295	51469670	51469826	8.88E-16	0.0000	0.133
ENSMUSG00000020397	Med7	11	+	46439805	46439975	46436973	46437191	46440562	46441527	2.26E-05	0.0172	0.128
ENSMUSG00000019961	Tmpo	10	-	91153277	91153397	91153084	91153180	91158747	91158845	8.17E-05	0.0377	-0.110
ENSMUSG00000021614	Vcan	13	-	89702917	89705797	89688284	89693501	89712204	89712498	2.79E-05	0.0197	-0.131
ENSMUSG00000031004	Mki67	7	-	135707360	135708320	135705609	135705785	135708765	135708811	3.81E-05	0.0241	-0.145
ENSMUSG00000034252	Senp6	9	+	80098885	80098906	80093536	80093641	80103693	80103764	8.11E-05	0.0377	-0.173
ENSMUSG00000022412	Mief1	15	+	80234681	80234780	80234079	80234181	80235804	80236040	5.41E-06	0.0061	-0.208
ENSMUSG00000047534	Mis18bp1	12	-	65161402	65162028	65158394	65158861	65172448	65172580	5.98E-05	0.0313	-0.220
ENSMUSG00000050619	Zscan29	2	-	121169198	121169403	121166043	121166718	121169809	121170268	5.34E-05	0.0300	-0.223
ENSMUSG00000047921	Trappc9	15	-	73042176	73042203	73031521	73031643	73050594	73050723	3.80E-08	0.0001	-0.238
ENSMUSG00000019813	Cep57l1	10	-	41728627	41728714	41723860	41723938	41729330	41729408	9.20E-05	0.0410	-0.266
ENSMUSG00000042446	Zmym4	4	-	126907085	126907236	126905871	126906147	126910789	126911002	8.40E-05	0.0380	-0.267
ENSMUSG00000072501	Phf20l1	15	+	66623760	66623883	66622760	66622817	66623991	66624099	5.90E-05	0.0313	-0.268
ENSMUSG00000003282	Plag1	4	-	3905446	3905805	3900995	3904947	3908905	3909002	6.50E-05	0.0323	-0.273
ENSMUSG00000049606	Zfp644	5	-	106695136	106695699	106666784	106666845	106696233	106696546	1.59E-05	0.0129	-0.277
ENSMUSG00000039382	Wdr45	X	+	7723564	7723753	7722249	7722378	7723848	7723920	1.76E-06	0.0027	-0.288
ENSMUSG00000038708	Golga4	9	+	118537241	118537325	118536758	118536943	118538876	118539024	8.04E-05	0.0377	-0.316
ENSMUSG00000055720	Ubl7	9	+	57911709	57911825	57911050	57911171	57912637	57912850	5.95E-05	0.0313	-0.345
ENSMUSG00000025920	Stau2	1	-	16231002	16231091	16228673	16229866	16345703	16346011	9.34E-08	0.0003	-0.378
ENSMUSG00000032228	Tcf12	9	-	72006700	72006762	72000415	72000518	72109675	72109748	3.45E-05	0.0223	-0.389
ENSMUSG00000031885	Cbfb	8	+	105202451	105202547	105171305	105171392	105215903	105217981	3.70E-06	0.0045	-0.394
ENSMUSG00000021098	4930447C04Rik	12	-	72915355	72915482	72913184	72913274	72916673	72916730	7.31E-06	0.0077	-0.408
ENSMUSG00000057469	E2f6	12	+	16813846	16813912	16811098	16811315	16816399	16816454	1.00E-10	0.0000	-0.418

ENSMUSG00000007812	Zfp655	5	+	145235305	145235391	145233440	145233602	145235736	145235863	5.07E-05	0.0290	-0.443
ENSMUSG00000097039	Pvt1	15	+	62244718	62244857	62242433	62242561	62249157	62249223	1.22E-05	0.0111	-0.446
ENSMUSG00000074643	Cpne1	2	-	156098229	156098372	156079338	156079464	156111827	156111909	1.44E-07	0.0004	-0.449
ENSMUSG00000062198	2700097O09Rik	12	-	55059374	55059578	55057241	55057352	55062987	55063109	1.61E-05	0.0129	-0.452
ENSMUSG00000021998	Lcp1	14	+	75176814	75176933	75136331	75136642	75198076	75198142	4.46E-05	0.0269	-0.461
ENSMUSG00000063063	Ctnna2	6	-	77914405	77914507	77845477	77845584	77979419	77979699	6.41E-05	0.0323	-0.475
ENSMUSG00000040312	Cchcr1	17	+	35517790	35517939	35517175	35517229	35518216	35518283	2.68E-05	0.0197	-0.483
ENSMUSG00000025217	Btrc	19	+	45406544	45406586	45363751	45363886	45471014	45471104	3.42E-06	0.0045	-0.560
ENSMUSG00000036278	Macrod1	19	+	7197701	7197736	7197531	7197613	7197912	7198061	1.21E-05	0.0111	-0.630
ENSMUSG00000074500	Zfp558	9	-	18461339	18461403	18459656	18459752	18467919	18468046	4.56E-05	0.0269	-0.637
ENSMUSG00000051331	Cacna1c	6	-	118741870	118741974	118734850	118735023	118742264	118742368	4.53E-07	0.0009	-0.644
ENSMUSG00000057363	Uxs1	1	-	43790463	43790550	43780064	43780169	43797244	43797305	1.39E-05	0.0120	-0.658
ENSMUSG00000049606	Zfp644	5	-	106695642	106695699	106666784	106666845	106696233	106696544	9.47E-07	0.0015	-0.662
ENSMUSG00000109284	B230311B06Rik	7	+	118594952	118595034	118592515	118592609	118596424	118596741	8.40E-07	0.0014	-0.689
ENSMUSG00000037236	Matr3	18	+	35562894	35563100	35562191	35562259	35571842	35572123	1.22E-15	0.0000	-0.743

A3SS

GeneID	geneSymbol	chr	strand	longExonStart	longExonEnd	shortES	shortEE	flankingES	flankingEE	P-Value	FDR	IncLevelDiffer
ENSMUSG00000022150	Dab2	15	+	6427330	6427765	6427520	6427765	6424634	6424697	1.35E-06	0.0024	0.797
ENSMUSG00000113204	Gm46430	13	-	74611263	74611387	74611263	74611321	74612723	74612948	1.90E-05	0.0222	-0.353
ENSMUSG00000039988	Ankrd13c	3	+	157972999	157975294	157975227	157975294	157972231	157972317	7.91E-07	0.0024	-0.451

A5SS

GeneID	geneSymbol	chr	strand	longExonStart	longExonEnd	shortES	shortEE	flankingES	flankingEE	PValue	FDR	IncLevelDifference
ENSMUSG00000042520	Ubap2l	3	-	90038814	90039016	90038847	90039016	90038369	90038465	0	0.0000	0.495
ENSMUSG00000031626	Sorbs2	8	+	45799709	45799801	45799709	45799798	45800964	45801011	1.76E-05	0.0019	0.402
ENSMUSG00000058594	Fbxo18	2	-	11769749	11769931	11769775	11769931	11767121	11767711	0	0.0000	0.377
ENSMUSG00000029587	Zfp12	5	+	143239953	143240150	143239953	143240080	143240319	143240415	0.000802416	0.0487	0.253

ENSMUSG00000031885	Cbfb	8	+	105202451	105202578	105202451	105202547	105215903	105217981	0	0.0000	0.245
ENSMUSG00000024012	Mtch1	17	-	29336166	29336311	29336217	29336311	29333957	29334005	0	0.0000	0.228
ENSMUSG00000026701	Prdx6	1	-	161250756	161251166	161251021	161251166	161249358	161249469	2.05E-06	0.0003	0.219
ENSMUSG00000001151	Pcnt	10	-	76380162	76380439	76380216	76380439	76379904	76380055	6.92E-06	0.0010	0.208
ENSMUSG00000029478	Ncor2	5	-	125028611	125028798	125028620	125028798	125026801	125027217	0.000106732	0.0092	0.191
ENSMUSG00000054252	Fgfr3	5	+	33721723	33721954	33721723	33721928	33722305	33722511	0.00011146	0.0092	0.190
ENSMUSG00000042489	Clspn	4	+	126573135	126573347	126573135	126573308	126574640	126574816	0.00086503	0.0498	0.187
ENSMUSG00000001120	Pcbp3	10	-	76785070	76785214	76785139	76785214	76781831	76781873	1.23E-09	0.0000	0.182
ENSMUSG00000049550	Clip1	5	-	123630224	123631169	123630452	123631169	123627293	123627410	0.000821203	0.0487	0.160
ENSMUSG00000001034	Mapk7	11	-	61493596	61493954	61493646	61493954	61492903	61493069	0.000108031	0.0092	0.152
ENSMUSG00000032280	Tle3	9	+	61394607	61394712	61394607	61394682	61401852	61402057	0.000782992	0.0487	0.136
ENSMUSG00000046711	Hmga1	17	+	27559532	27559703	27559532	27559670	27560927	27561011	0.000419244	0.0290	0.104
ENSMUSG00000029687	Ezh2	6	-	47576535	47576664	47576562	47576664	47558177	47558294	1.13E-05	0.0014	0.100
ENSMUSG00000038429	Usp5	6	-	124818474	124818666	124818543	124818666	124817887	124818031	0.000225195	0.0173	0.092
ENSMUSG00000025873	Faf2	13	+	54641418	54641553	54641418	54641496	54645244	54645321	2.68E-06	0.0004	-0.106
ENSMUSG00000030960	Eef1akmt2	7	-	132850590	132850709	132850594	132850709	132837099	132837207	0.000403104	0.0290	-0.131
ENSMUSG00000030516	Tjp1	7	-	65299669	65299813	65299708	65299813	65296707	65297667	0.000167604	0.0134	-0.151
ENSMUSG00000026349	Ccnt2	1	+	127797840	127798196	127797840	127797886	127799389	127799553	0.000420127	0.0290	-0.166
ENSMUSG00000024220	Zfp523	17	+	28201260	28202348	28201260	28201440	28202434	28202598	0.000703279	0.0456	-0.183
ENSMUSG00000046897	Zfp740	15	+	102204570	102205086	102204570	102204883	102207770	102207920	3.74E-05	0.0037	-0.204
ENSMUSG00000022992	Kansl2	15	-	98524458	98524712	98524560	98524712	98520317	98520425	4.20E-09	0.0000	-0.210
ENSMUSG00000040124	Gorab	1	-	163396807	163397169	163396811	163397169	163394590	163394692	1.95E-05	0.0020	-0.226
ENSMUSG00000038056	Kmt2c	5	-	25281660	25281803	25281672	25281803	25281231	25281400	1.17E-05	0.0014	-0.252
ENSMUSG00000026005	Rpe	1	+	66701616	66701648	66701616	66701636	66706445	66706525	8.44E-05	0.0080	-0.255
ENSMUSG00000037846	Rtkn2	10	+	67987306	67987512	67987306	67987503	67997564	67997623	1.67E-05	0.0019	-0.260
ENSMUSG00000053477	Tcf4	18	+	69677966	69678129	69677966	69678117	69681568	69681798	1.76E-08	0.0000	-0.347
ENSMUSG00000022263	Trio	15	-	27742371	27742510	27742383	27742510	27741251	27741279	2.46E-10	0.0000	-0.371
ENSMUSG00000067367	Lyar	5	+	38223133	38223231	38223133	38223227	38224626	38224797	3.22E-15	0.0000	-0.420

ENSMUSG00000091625	Lsm5	6	-	56703313	56703409	56703317	56703409	56702095	56702168	0.000449959	0.0301	-0.491
ENSMUSG00000040687	Madd	2	-	91158404	91158488	91158416	91158488	91157984	91158143	5.59E-10	0.0000	-0.529
ENSMUSG00000024985	Tcf7l2	19	+	55910578	55910681	55910578	55910669	55912558	55912645	0	0.0000	-0.576
ENSMUSG00000025656	Arhgef9	X	-	95165588	95165970	95165592	95165970	95130835	95131015	4.66E-15	0.0000	-1.000

RI

GeneID	geneSymbol	chr	strand	riExonStart	riExonEnd	upstreamES	upstreamEE	downstreamES	downstreamEE	PValue	FDR	IncLevelDifference
ENSMUSG00000024170	Telo2	17	-	25101639	25103771	25101639	25101704	25103579	25103771	1.08E-06	0.0016	0.511
ENSMUSG00000071337	Tia1	6	+	86423605	86424420	86423605	86423671	86424344	86424420	2.30E-06	0.0022	0.477
ENSMUSG00000020692	Nle1	11	-	82902994	82904136	82902994	82903133	82904089	82904136	8.71E-05	0.0267	0.302
ENSMUSG00000039205	Ciz1	2	+	32370863	32371408	32370863	32371101	32371248	32371408	2.88E-05	0.0132	0.223
ENSMUSG00000079487	Med12	X	+	101293957	101294175	101293957	101294012	101294087	101294175	3.19E-05	0.0132	0.181
ENSMUSG00000026848	Tor1b	2	+	30953751	30955953	30953751	30954008	30955777	30955953	1.20E-05	0.0070	-0.302
ENSMUSG00000053453	Thoc7	14	-	13953410	13954656	13953410	13953538	13954565	13954656	9.21E-05	0.0267	-0.333
ENSMUSG00000021235	Coq6	12	+	84370255	84371558	84370255	84371031	84371450	84371558	5.44E-05	0.0197	-0.412
ENSMUSG00000026349	Ccnt2	1	+	127791604	127795800	127791604	127791733	127795254	127795800	5.37E-06	0.0039	-0.595
ENSMUSG00000054199	Gon4l	3	+	88898104	88899074	88898104	88898360	88898889	88899074	5.07E-10	0.0000	-0.725

MXE

GeneID	geneSymbol	chr	strand	1stExonStart	1stExonEnd	2ndExonStart_0base	2ndExonEnd	upstreamES	upstreamEE	downstreamES	downstreamEE	PValue	FDR	IncLevelDifference
ENSMUSG00000061983	Rps12	10	-	23785575	23785677	23785901	23786004	23785182	23785273	23786624	23786802	1.67E-10	0.0000	0.702
ENSMUSG00000051331	Cacna1c	6	-	118741870	118741974	118742264	118742368	118734850	118735023	118751368	118751565	2.49E-07	0.0003	0.504
ENSMUSG00000031309	Rps6ka3	X	+	159278109	159278203	159279166	159279203	159276275	159276336	159285265	159285322	2.22E-05	0.0228	0.277
ENSMUSG00000031885	Cbfb	8	+	105178592	105178709	105202451	105202547	105171305	105171392	105215903	105217981	1.19E-08	0.0000	0.127

chr: chromosome
+/-: plus / minus strand
SE: Skipped exon
A3SS: Alternative 3' splice site
A5SS: Alternative 5' splice site

RI: Retained intron
MXE: Mutually exclusive exon
exonStart: Start position of alternative exon in chromosome
exonEnd: End position of alternative exon in chromosome
upstreamES: Constitutive up stream exon start site in chromosome
upstreamEE: Constitutive upstream exon end site in chromosome
downstreamES: Constitutive downstream exon start site in chromosome
downstreamEE: Constitutive downstream exon end site in chromosome
FDR: False discovery rate
IncLevelDiffer: Inclusion level difference

Table S5. Rbm42-regulated alternative splicing events assocciated with Hippo pathway

GeneID	geneSymbol	splicing type	alternative exon loci	P-value	FDR	IncLevelDiffer
ENSMUSG00000020271	Fbxw11	SE	chr11(+):32708187-32708250	3.42E-06	0.0045	0.259
ENSMUSG00000063063	Ctnna2	SE	chr6(-):77914405-77914507	6.41E-05	0.0323	-0.475
ENSMUSG00000025217	Btrc	SE	chr19(+):45406544-45406586	3.42E-06	0.0045	-0.560
ENSMUSG00000024985	Tcf7l2	A5SS	chr19(+):55910578-55910681	0	0.0000	-0.576

chr: chromosome
+/-: plus / minus strand
SE: Skipped exon
A5SS: Alternative 5' splice site
FDR: False discovery rate
IncLevelDiffer: Inclusion level difference
